# A Versatile System of Solvent, Catalyst, and Ligand for Challenging Biphenyl Synthesis Through Suzuki‐Miyaura Reactions

**DOI:** 10.1002/chem.202501789

**Published:** 2025-11-26

**Authors:** Mahmoud K. Al‐Joumhawy, Jui‐Chi Chang, Detlef Gabel

**Affiliations:** ^1^ Department of Chemistry Faculty of Sciences Yarmouk University Irbid Jordan; ^2^ School of Science Campus Ring 1 Constructor University Bremen Bremen Germany

**Keywords:** davephos, electron‐deficient substrates, n‐methylpyrrolidone, suzuki‐Miyaura cross‐coupling, tris(dibenzylideneacetone)dipalladium(0)

## Abstract

Suzuki‐Miyaura cross‐coupling reactions are difficult when both reaction partners (boronic acid and halide) are electron‐poor. We describe here a system of solvent, catalyst, and ligand with which also electron‐poor substrates react, without the necessity to try several conditions. The system consists of N‐methylpyrrolidone as solvent, tris(dibenzylidenaceton)dipalladium(0) as catalyst, CuI as cocatalyst, and Davephos as ligand. This combination yields little or no Ullmann product, and the desired cross‐coupling product is in moderate to excellent yields.

## Graphical abstract

Suzuki‐Miyaura cross‐coupling reactions with electron‐deficient haloarenes and electron‐deficient areneboronic acids proceed with moderate to excellent yields in N‐methylpyrrolidone as solvent, Pd(dba)_3_ as catalyst, CuI as cocatalyst, and Davephos as ligand.

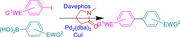

## 1 Introduction

Suzuki‐Miyaura cross‐coupling (SMC) is a very versatile method to connect boronic acid compounds with halo compounds, forming a C‐C bond. Since its conception, it has attracted chemists due to the ease of preparation of its reaction partners, their stability, and the large number of unrelated functional groups compatible with this reaction. For the reaction, a boronic acid component (either as such or as a suitable derivative) and a halo compound are required. As catalysts, Pd compounds with suitable ligands have been employed. In most cases, the reaction proceeds smoothly, quickly, and with good purity.

In some cases, SMC reactions face difficulties. This seems to be the case with either sterically demanding reaction partners or reactions of partners with strongly electron‐withdrawing substituents on one or both of the partners. Thus, 2‐nitrohaloarenes and 2‐nitrophenylboronic acids do not react readily, and a considerable amount of homo‐coupling (Ullmann product) is observed [1]. For 3‐ and 4‐nitrohaloarenes and 3‐ and 4‐nitrophenylboronic acids, the yields are not much better. Steric hindrance is another confounding factor, sometimes completely preventing the formation of the desired coupling product [2].

In our own work, we have used SMC to react iodo‐dodecaborate (B_12_H_11_I[2‐]) and aryl‐, alkyl‐, and vinylboronic acids to produce B‐C bonds between the dodecaborate and a large variety of organic moieties [3]. We found that with this particular halo compound, the choice of Pd catalyst, ligand, and solvent was of great importance. With the wrong combination, reactions did not proceed at all or were extremely sluggish or resulted in the formation of by‐products. Surprising was the observation that electron‐poor boronic acids required N‐methylpyrrolidone (NMP) as a solvent; on the other hand, electron‐rich boronic acids reacted only in CD_3_CN. In both cases, Davephos was the best ligand; as a Pd source, tris(dibenzylideneacetone)dipalladium(0) (Pd_2_(dba)_3_) was used.

Before, we had investigated the electron distribution in the per‐iodinated dodecaborate B_12_I_12_[2‐][4]. In this compound, the dodecaborate unit withdraws the equivalent of a full electron from the combined iodine atoms. With this in mind, we hypothesized that perhaps similar reaction conditions as the ones for the reaction with B_12_H_11_I[2‐] might also allow such SMC reactions to proceed for which standard conditions were not adequate.

Some reactions between electron‐poor aromatic boronic acids and electron‐poor aryl halides have been described before [1,2, 5, 6, 7, 8, 9, 10, 11]. There was no systematic comparison of the different reaction conditions (solvent, catalyst, ligand), and the transfer to new reactions does not seem to be straightforward at all. With a success in the strategy developed for reacting electron‐poor boronic acids with B_12_H_11_I[2‐], a more general approach to successful SMC with demanding substrates might be achievable.

## 2 Results and Discussion

With the conditions developed before [3] for the reaction of B_12_H_11_I[2‐], we reacted six different aryl halides with a total of eight arylboronic acids. The reactants are shown in Table 1. As a condition, we chose 2.5 mol% Pd_2_(dba)_3_, 5 mol% Davephos, 2.5 equivalents of KOH, and 2 mL NMP for 0.5 mmol haloarene, reaction temperature 100°C (oil bath), reaction time 2 h, under a flow of nitrogen. The boronic acids were used in 1.5‐fold excess.

**TABLE 1:** SMC reactions performed.

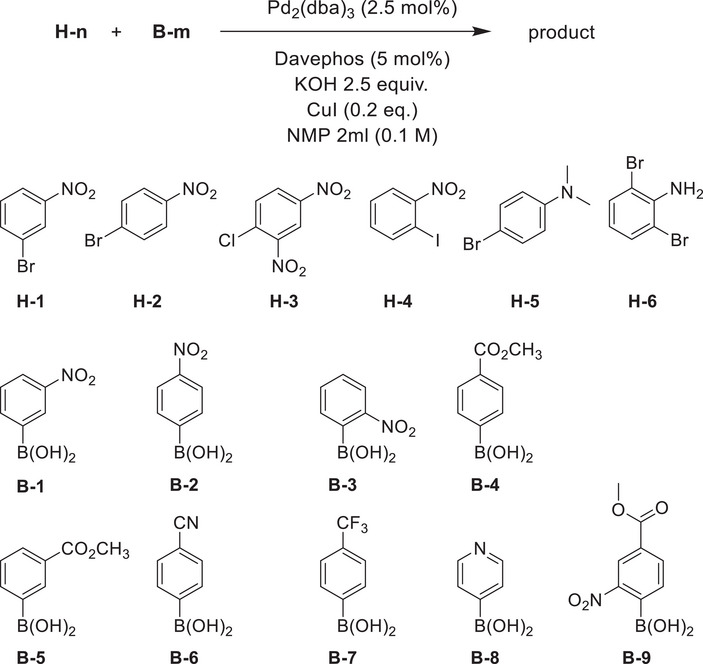

Entry	H partner	B‐partner	Yield / Ullmann	Structure	Literature Suzuki/ different (yield %)
1	H‐1	B‐1	76 / <10	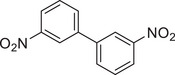	S: [1] (38)
2	H‐1	B‐2	80 / <5	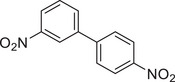	S: [10] (85)
3	H‐1	B‐3	77 / <9	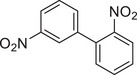	S: [1] (10)
4	H‐1	B‐4	84 / 0	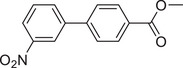	S: [20] (16)
5	H‐1	B‐5	83 / 0	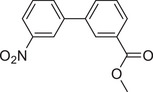	S: [20] (95)
6	H‐1	B‐6	90 / 0	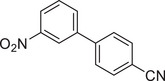	S:[9] (95)
7	H‐1	B‐7	91 / 0	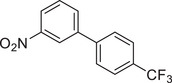	S: [21] (31)
8	H‐1	B‐8	73 / <10	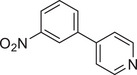	S: [7] (not given)
9	H‐2	B‐1	48 / 10	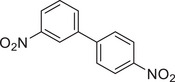	S: [5] (94)
10	H‐2	B‐2	95 / 0	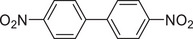	S: [6] (100) [1] (12)
11	H‐2	B‐3	66 / 12	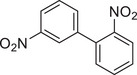	(same compound as entry 3)
12	H‐3	B‐1	17 / <5	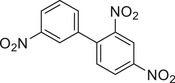	S: [22] (71)
13	H‐3	B‐2	21 / 20^[^ ^a^ ^]^	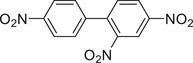	(prepared by Negishi coupling: [23])
14	H‐3	B‐3	38 / 13	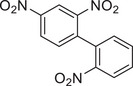	(commercially available, but no characterization data published)
15	H‐3	B‐4	46 / <10	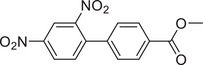	S (phenyl ester): [24] (75) ethyl ester by Negishi coupling: [23]
16	H‐3	B‐5	45 / <10	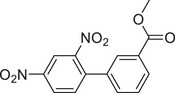	
17	H‐4	B‐1	65 / <5	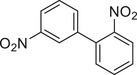	(same compound as entry 3)
18	H‐4	B‐3	88 / 2	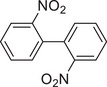	S: [1] (27)
19	H‐5	B‐1	90 / 0	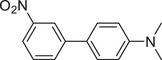	S: [25] (92)
20	H‐6	B‐9^[^ ^b^ ^]^	54 / 5	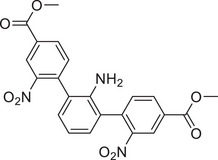	S: [2] (88)

^[a]^ obtained as 1:1 mixture with the Ullmann product, which could not be separated.

^[b]^ 1.5 equivalents were used except for **B‐9**, of which two equivalents were used.

The halides (numbered **H‐**1 through **H‐6**) used were electron‐deficient, with Cl, Br, and I as halides and one or two nitro substituents. The halides **H‐5** and **H‐6** were included as examples of electron‐rich aryl halides with one or two halogen atoms. All the boronic acids (numbered **B‐1** through **B‐9**) were also electron‐deficient, with the exception of **B‐8**.

In every single pair of halides and boronic acid that we took to reaction, the reaction product could be obtained and isolated. This includes the coupling with *o*‐nitrophenylboronic acid (for difficulties with such compounds, see example [2]). It also includes one example of an electron‐rich boronic acid (**B‐8**).

Our intention was to see whether the conditions successful for B_12_H_11_I[2‐] would also be adequate for electron‐poor halides reacting with electron‐poor boronic acids. We have therefore not investigated whether catalyst load could be lower and other parameters could be optimized. It is remarkable that the many different conditions used in the referenced compounds of Table 1 could be replaced by a single standard set of catalyst, ligand, solvent, and temperature.

In Heck reactions, a positive effect of NMP as a solvent had been observed before [12]. It was postulated there that NMP complexes (via the N atom) with Pd^2+^. Alternatively, the interaction of NMP with Fe^3+^‐catalyzed cross‐couplings has been postulated to involve the O atom [13]. It might be envisaged that similar interactions might play a role in the reaction of electron‐poor boronic acid substrates, stabilizing one or both of the intermediates resulting from oxidative addition or transmetalation. For aryl‐aryl SMC, NMP has been described as a suitable solvent;[14] in general, solvents can have a profound influence on SMC [15]. While the role of Cu(I) in this particular reaction is not clear, it has been reported that it supports Sonogashira coupling [16].

The synthesis of several 2‐nitro and 2, 2’‐binitrodiphenyls has been attempted by others before [1, 17]. Yields were poor, and Ullmann‐type products resulting from homo‐coupling—which usually results from homo‐coupling of haloarenes or boronic acids—were observed. In our case, Ullmann products were in many cases not detected (they were found in entries 3, 12, 17, and 18 of Table 1 in less than 20%). Thus, the coupling between **H‐1** and **B‐3** proceeded to 77% yield, whereas the literature gives 10% yield [1]. Equally, we achieved a yield of 76% between **H‐1** and **B‐1** and of 88% between **H‐4** and **B‐3**, whereas the literature reports 38% resp. 30% [1]. When using microwave heating and higher temperatures, literature reports that yields were low, and considerable reaction of the halide with the solvent was observed [17]. Interestingly, the literature reports on obtaining 2‐nitrobiphenyls (but not 4‐nitrobiphenyls) from 2‐nitrofluorobenzene and phenylboronic acid;[18] in that case, the complexation of the 2‐nitro group to Pd after oxidative insertion into the C‐F bond seemed to be essential. Under the conditions developed here, complexation of the nitro group does not seem to be of great importance.

In view of the many examples in the literature (among them the synthesis of some of the compounds of Table 1 produced by others before), reaction conditions for each reacting pair of halides and boronic acid can probably be optimized. As an illustration, literature data on the yield of the product between **H‐2** and **B‐2** have been reported as 100% (using a nanocatalyst in deep eutectic solvent)[6] and 12% (Pd(OAc)_2_ with tetrabutylammonium bromide and KH_2_PO_4_ in DMF)[1]. The yield using the standard conditions of this work (95%) compared favorably to that obtained with the nanocatalyst in eutectic solvent. For the product between **H‐4** and **B‐3** (entry 18 of Table 1), a Pd pincer complex was reported to yield 80% of the product, similar to the results obtained here (88%) [19].

We have not studied the reason for the performance of the combination of catalyst, ligand, and solvent here. For the cross‐coupling with iodododecaborate, we have concluded that the amino group on DavePhos mediates the docking of the activated boronic acid to the catalytic system through a hydrogen bond [3]. Additionally, NMP's role as a solvent is well‐established, and its coordination behavior with palladium centers can affect the reaction's mechanism and selectivity.

The conditions for the cross‐coupling reaction did not work for free carboxylic acids as partners. We attribute this to competition between the carboxylic acid and the Davephos ligand with the Pd catalyst. The methyl esters reacted, however, without problems in good yields. Diboronic acids also showed difficulties.

While optimization is certainly possible for each individual reaction, it might be doubtful whether such optimized conditions might be applicable for an arbitrary, so far untried combination of halide and boronic acid. We therefore suggest using the reaction conditions described here as a starting point for further, individual optimization of reaction conditions and using the reaction conditions especially also when the initial yield of a reaction is of lesser concern.

## 3 Conclusion

With the choice of NMP as solvent, Pd_2_(dba)_3_ as catalyst, CuI as cocatalyst, and Davephos as ligand, Suzuki‐Miyaura cross‐couplings also proceed when both the haloarene and the arylboronic acid are electron‐deficient. Although not tested extensively, the reaction conditions seem also appropriate for electron‐rich areneboronic acids and therefore offer a reliable system for such cross‐couplings with untested substrates.

## 4 Experimental Section

### General procedure for the palladium‐catalyzed Suzuki‐type cross‐coupling of haloarenes:

To a dry 10 mL round‐bottom flask equipped with a magnetic stir bar, haloarenes (0.5 mmol), Pd_2_(dba)_3_ (2.5 mol%, 0.0125 mmol), Davephos (5.0 mol%, 0.025 mmol), KOH (1.25 mmol), boronic acid derivative (1.5 eq., 0.75 mmol), CuI (0.2 eq., 0.025 mmol), and NMP (2 mL) were added. The reaction mixture was purged by N_2_ flow for 30 s and connected to a condenser under continuous N_2_ flow. The resulting mixture was stirred for 3 h at 90°C. The mixture was filtered through Celite. The filtrate was then evaporated to dryness under reduced pressure. The crude product was purified by chromatography on silica gel (gradient elution: hexane/EtOAc:hexane 1:1) to afford the desired product.

## Conflicts of Interest

The authors declare no conflicts of interest.

## Supporting information




**Supporting File 1**: Detailed synthesis description and spectral characterization.
