# Supplementary material for: A Versatile System of Solvent, Catalyst, and Ligand for Challenging Biphenyl Synthesis Through Suzuki‐Miyaura Reactions
Source: Chemistry. 2025 Nov 26;31(72):e01789. doi: 10.1002/chem.202501789 (PMC12731529; doi:10.1002/chem.202501789)
Supplement: Supplementary file 1 — Supporting File 1: Detailed synthesis description and spectral characterization. [file CHEM-31-e01789-s001.pdf]

A Versatile System of Solvent, Catalyst, and Ligand for Challenging Suzuki-Miyaura Reactions

Mahmoud K. Al-Joumhawy†, Jui-Chi Chang‡, Detlef Gabel‡ *

† Department of Chemistry, Faculty of Science, Yarmouk University, Irbid 21163, Jordan. e-mail: mjomhawi@yu.edu.jo

‡ School of Science, Constructor University Bremen gGmbH, Campus Ring 1, D-28759 Bremen. Germany

* e-mail: dgabel@constructor.university

SUPPORTING INFORMATION

Table of Contents

General Information 1

General procedure for the palladium-catalyzed Suzuki-type cross coupling of haloarenes: 2

Synthesis and characterization of cross coupling products:...3

NMR spectra of coupling products 9

General Information

Chemicals: The starting material $Pd_2(dba)_3$, DavePhos, boronic acids, substituted phenylboronic acids, boronic acid pinacol esters, N-methyl-2-pyrrolidone (NMP), and $CDCl_3$/DMSO-d_6 were purchased from Sigma Aldrich. All reagents were used as received without purification unless noted otherwise. Purification of reaction products was carried out by column chromatography on silica gel 60 (200-30 mesh). Anhydrous solvents were prepared by passing through activated CaH_2 and stored over 3 Å molecular sieves. Chemical yields refer to isolated products after chromatography.

Reaction Conditions: Glassware for all cross coupling reactions was dried at 150 °C for 12 h and allowed to cool under nitrogen atmosphere. All experiments were carried out under nitrogen atmosphere; see the procedure for details.

Characterization: Thin-layer chromatography (TLC) was carried out using silica gel 60, F254 with a thickness of 0.2 mm. TLC samples for borane-containing compounds were stained with CAM solution. Column chromatography was performed on silica gel 60 (0.07-0.2 mesh). NMR spectra were recorded on a JEOL 400 spectrometer (^{1}H NMR 400.13 MHz, ^{13}C NMR 100.0 MHz, ^{11}B NMR 128.38 MHz) at 25 °C. Chemical shifts are given in ppm. ^{1}H NMR and ^{13}C NMR spectra were referenced using the solvent signals (^{1}H: CDCl3 = 7.26 ppm, $CD_3S(O)CD_3$ = 2.5 ppm, ^{13}C{1 H}: Chloroform-d = 77.16), DMSO-d6 = 39.5 ppm). Data are reported as follows: Chemical shift in ppm, multiplicity (s = singlet, d = doublet, t = triplet, q = quartet, m = multiplet, dd = doublet of doublets, etc.), coupling constant J in Hz, integration, and (where applicable) interpretation.

General procedure for the palladium-copper-catalyzed Suzuki-type cross coupling of haloarenes:

To a dry 10 mL round bottom flask equipped with a magnetic stir bar, haloarenes (0.5 mmol), $Pd_2(dba)_3$ (2.5 mol%, 0.0125 mmol), Davephos, (5.0 mol%, 0.025 mmol), KOH (1.25 mmol), boronic acid derivative (1.5 eq., 0.75 mmol), CuI (0.2 eq., 0.025 mmol) and NMP (2 ml) were added. The reaction mixture was purged by N_2 flow for 30 seconds and connected to a condenser under continuous N_2 flow. The resulting mixture was stirred for 3 h at 90 °C. The mixture was filtered through celite, washing the celite with DCM. The filtrate was then evaporated to dryness under reduced pressure. The crude product was purified by chromatography on silica gel (gradient elution: hexane/ EtOAc:hexane 1:1) to afford the desired product.

Synthesis and characterization of cross coupling products:

S1: 3,3'-dinitro-1,1'-biphenyl (Entry 1-Table 1)

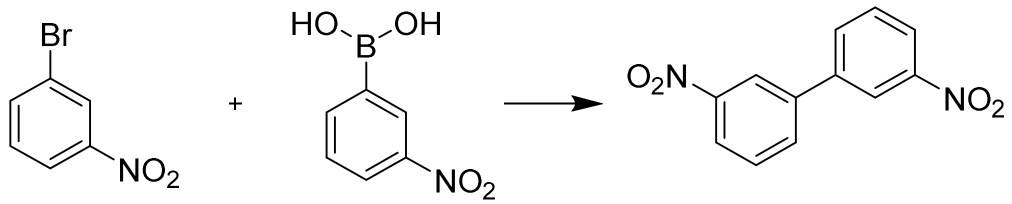

^{1}H NMR (400 MHz, Chloroform-*d*) δ 7.6 – 7.8 (t, *J* = 8.9 Hz), 7.9 – 8.0 (d, *J* = 9.1 Hz), 8.2 – 8.4 (d, *J* = 9.8 Hz), 8.5 – 8.6 (m) .

^{13}C NMR (125 MHz, Chloroform-*d*) δ 121.7, 124.1, 129.2, 131.9, 141.0, 147.4, 219.7.

S2: 3,4'-dinitro-1,1'-biphenyl (Entry 2-Table 1)

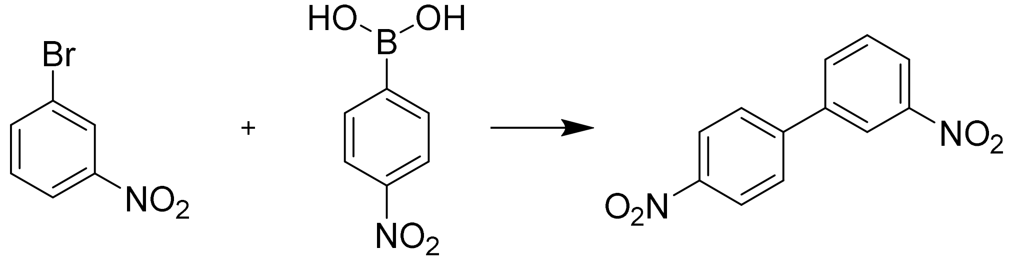

^{1}H NMR (400 MHz, DMSO-*d*6) δ 7.8 – 7.8 (td, *J* = 8.0, 5.3 Hz), 8.0 – 8.1 (d), 8.2 – 8.3 (m), 8.3 – 8.3 (d), 8.5 – 8.6 (dt, *J* = 4.3, 2.0 Hz).

^{13}C NMR (101 MHz, DMSO-*d*6) δ 122.4, 124.1, 124.7, 129.0, 131.3, 134.4, 139.9, 144.6, 147.9, 149.0.

S3: 2,3'-dinitro-1,1'-biphenyl (Entry 3-Table 1)

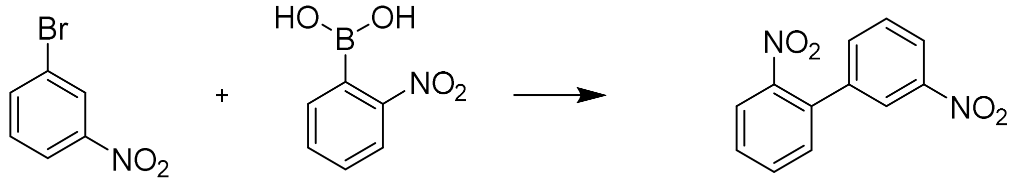

^{1}H NMR (400 MHz, Chloroform-*d*) δ 8.5 – 8.5 , 8.3 – 8.2 (dd, *J* = 5.5, 2.9 Hz), 8.2 – 8.2 , 8.0 – 8.0 (d, *J* = 8.5 Hz), 7.7 – 7.7 (m), 7.7 – 7.6 (d, *J* = 7.4 Hz), 7.5 – 7.4 (m).

^{13}C NMR (101 MHz, Chloroform-*d*) δ 123.2, 123.2, 124.8, 129.6, 129.7, 132.1, 133.2, 134.2, 139.5, 148.4, 148.7.

S4: methyl 3'-nitro-[1,1'-biphenyl]-4-carboxylate (Entry 4-Table 1)

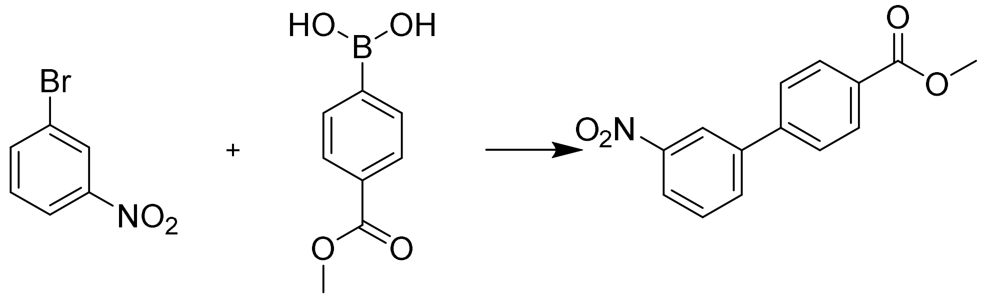

^{1}H NMR (400 MHz, Chloroform-*d*) δ 8.5 – 8.5 (t, *J* = 2.0 Hz), 8.3 – 8.2 (m, *J* = 8.2, 2.2, 0.9 Hz), 8.2 – 8.1 (m), 8.0 – 7.9 (m), 7.7 – 7.7 (m), 7.7 – 7.6 (m), 4.0 – 3.9 (s).

^{13}C NMR (101 MHz, Chloroform-*d*) δ 52.6, 120.1, 126.9, 129.0, 130.6, 131.2, 132.0, 133.5, 135.8, 141.5, 147.3, 148.9, 166.2. .

S5: methyl 3'-nitro-[1,1'-biphenyl]-3-carboxylate (Entry 5-Table 1**)**

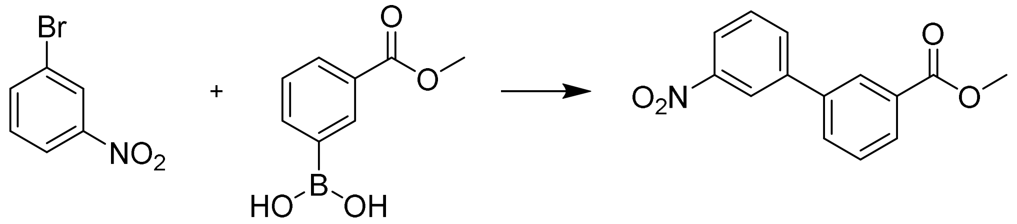

^{1}H NMR (400 MHz, CHLOROFORM-*D*) δ 8.46 (t, *J* = 2.1 Hz, 1H), 8.33 – 8.27 (m, 1H), 8.22 (dt, *J* = 8.2, 1.5 Hz, 1H), 8.09 (dt, *J* = 7.7, 1.5 Hz, 1H), 7.98 – 7.92 (m, 1H), 7.81 (dt, *J* = 7.8, 1.5 Hz, 1H), 7.68 – 7.52 (m, 2H), 7.41 (q, *J* = 3.2 Hz, 1H), 4.01 – 3.92 (m, 3H).

^{13}C NMR (101 MHz, CHLOROFORM-*D*) δ 165.01, 158.15, 148.30, 137.96, 135.33, 131.89, 129.70, 127.46, 127.39, 124.65, 122.77, 120.32, 52.87.

S6: 3'-nitro-[1,1'-biphenyl]-4-carbonitrile (Entry 6-Table 1)

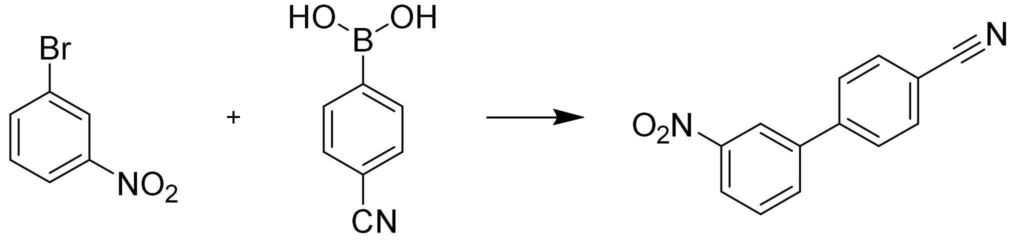

^{1}H NMR (400 MHz, CHLOROFORM-*D*) δ 8.28 (t, *J* = 1.9 Hz, 1H), 8.13 – 8.06 (m, 1H), 7.82 – 7.75 (m, 1H), 7.65 – 7.54 (m, 4H), 7.51 (t, *J* = 8.0 Hz, 1H).

^{13}C NMR (101 MHz, CHLOROFORM-*D*) δ 148.82, 142.19, 141.36, 133.24, 130.79, 130.46, 130.15, 127.62, 126.25, 126.22, 126.18, 126.14, 125.48, 122.99, 122.77, 122.14.

S7: 3-nitro-4'-(trifluoromethyl)-1,1'-biphenyl (Entry 7-Table 1)

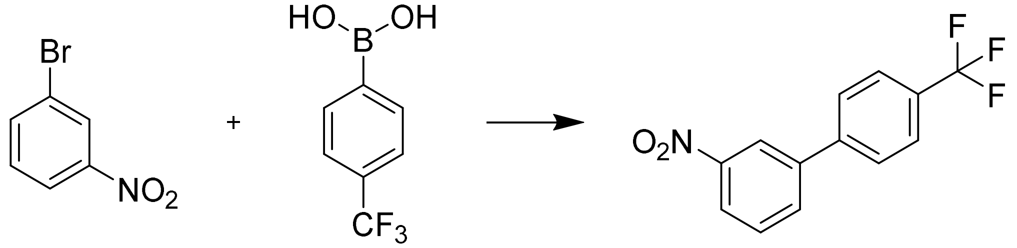

^{1}H NMR (400 MHz, Chloroform-*d*) δ 8.5 – 8.4 (m), 8.3 – 8.2 (m), 8.0 – 7.9 (m), 7.7 – 7.7 (m), 7.7 – 7.6 (m).

^{13}C NMR (101 MHz, CHLOROFORM-*D*) δ 148.84, 142.21, 141.43, 133.25, 130.84, 130.51, 130.15, 128.48, 127.65, 126.24, 126.20, 125.45, 123.02, 122.74, 122.20, 77.46, 77.15, 76.83.

S8: 4-(3-nitrophenyl)pyridine (Entry 8-Table 1)

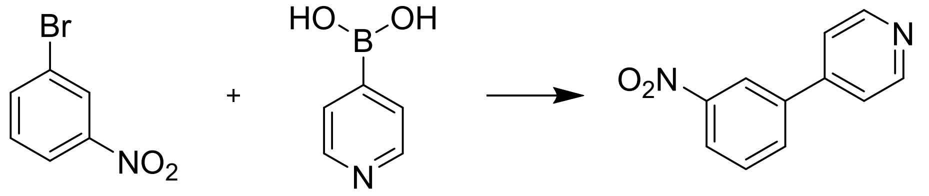

^{1}H NMR (400 MHz, Chloroform-*d*) δ 8.5 – 8.5 , , 8.3 – 8.3 (d, *J* = 8.2 Hz), 8.0 – 7.9 (d, *J* = 7.8 Hz), 7.7 – 7.7 (d, *J* = 7.1 Hz), 7.3 – 7.2 , .

^{13}C NMR (101 MHz, Chloroform-*d*) δ 123.0, 124.0, 129.1, 131.8, 133.2, 134.0, 144.6, 147.7, 148.6.

S9: 3,4'-dinitro-1,1'-biphenyl (Entry 9-Table 1)

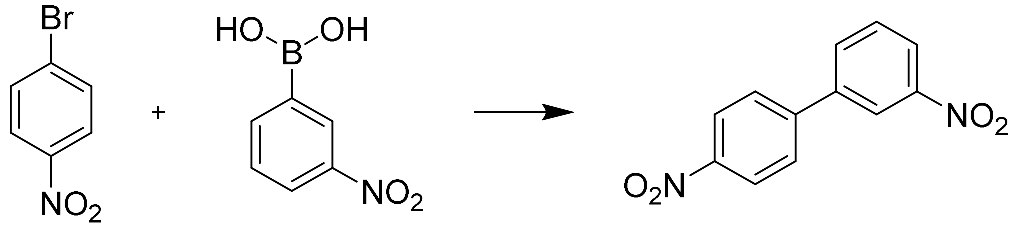

^{1}H NMR (400 MHz, DMSO-*d*6) δ 8.6 – 8.5 , , 8.4 – 8.3 (m), 8.3 – 8.2 (m), 8.1 – 8.1 (t), 7.9 – 7.8 .

^{13}C NMR (101 MHz, DMSO-*d*6) δ 122.4, 124.2, 124.8, 129.0, 131.4, 134.4, 140.0, 144.7, 147.9, 149.0.

S10: 4,4'-dinitro-1,1'-biphenyl (Entry 10-Table 1)

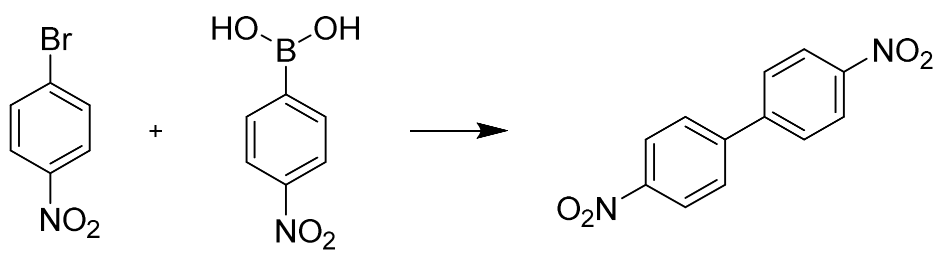

^{1}H NMR (400 MHz, DMSO-*d*6) δ 8.4 – 8.3 (d, *J* = 8.7 Hz), 8.1 – 8.0 (d, *J* = 8.6 Hz).

^{13}C NMR (101 MHz, DMSO-*d*6) δ 40.0, 124.8, 129.2, 144.6, 148.1.

S11: 2,4'-dinitro-1,1'-biphenyl (Entry 11-Table 1)

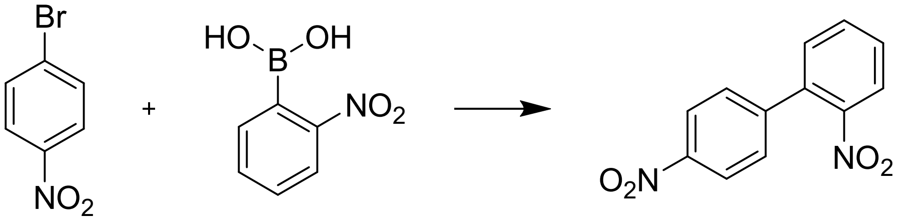

^{1}H NMR (500 MHz, Chloroform-*d*) δ 8.2 – 8.1 (m, 2H), 8.0 – 7.9 (dd, *J* = 7.4, 2.2 Hz, 1H), 7.9 – 7.9 (dd, *J* = 7.4, 2.1 Hz, 1H), 7.7 – 7.7 (m, 2H), 7.7 – 7.7 (m, 1H), 7.7 – 7.6 (td, *J* = 7.4, 2.1 Hz, 1H).

^{13}C NMR (101 MHz, Chloroform-*D*) δ 77.2, 124.0, 124.9, 129.1, 129.7, 131.8, 133.2, 134.6, 144.6, 147.7, 148.6.

S12: 2,3',4-trinitro-1,1'-biphenyl (Entry 12-Table 1)

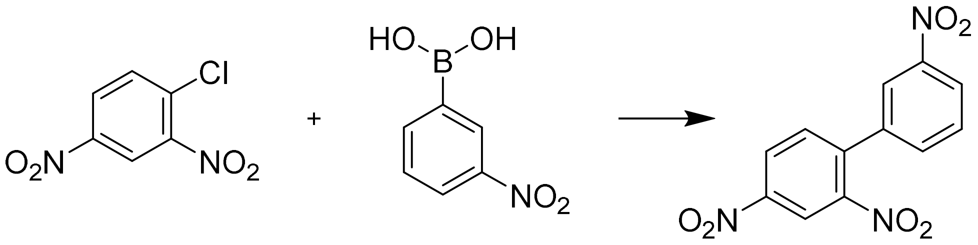

^{1}H NMR (400 MHz, Chloroform-*d*) δ 8.9 – 8.8 , , 8.6 – 8.5 (dd, *J* = 8.4, 2.2 Hz), 8.4 – 8.3 (d, *J* = 8.0 Hz), 8.3 – 8.2 , , 7.7 – 7.6 (m).

^{13}C NMR (101 MHz, Chloroform-d) δ 77.1, 120.4, 123.0, 124.4, 127.3, 130.2, 133.5, 133.8, 137.2, 140.0, 147.8, 148.5, 148.8.

S13: 2,4,4'-trinitro-1,1'-biphenyl (Entry 13-Table 1)

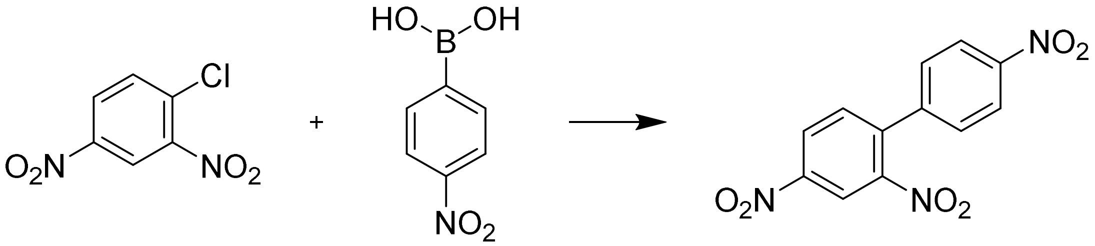

^{1}H NMR (400 MHz, Chloroform-*d*) δ 8.5 – 8.4 (d, *J* = 9.9 Hz), 8.3 – 8.2 (m), 7.6 – 7.4 (m), 7.4 – 7.3 (d, *J* = 9.1 Hz), 7.2 – 7.1 (dd, *J* = 8.5, 4.5 Hz).

S14: 2,2',4-trinitro-1,1'-biphenyl (Entry 14-Table 1)

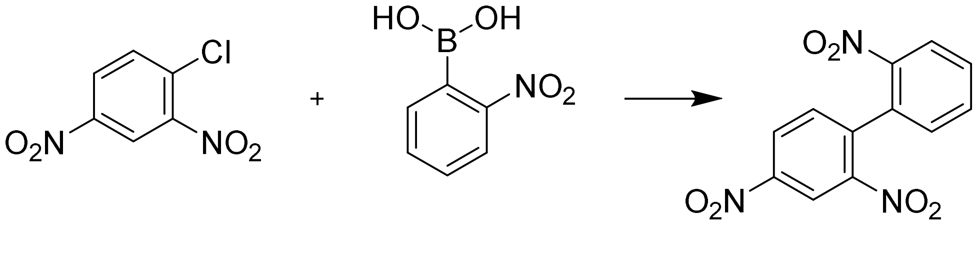

1H-NMR (400 MHz, Chloroform-d) δ 9.1 – 9.0 (d, J = 2.3 Hz), 8.6 – 8.5 (m), 8.3 – 8.3 (dd, J = 8.2, 1.3 Hz), 7.8 – 7.7 (td, J = 7.5, 1.1 Hz), 7.7 – 7.7 (ddd, J = 8.6, 7.8, 1.3 Hz), 7.6 – 7.5 (d, J = 8.4 Hz), 7.3 – 7.3 (dd, J = 7.6, 1.5 Hz).

^{13}C NMR (101 MHz, Chlorofom-*d*) δ 77.1, 120.4, 125.4, 127.7, 130.4, 132.3, 132.4, 134.2, 140.8, 146.6, 147.3, 147.7.

Elemental analysis C: 49.72%, H: 2.55%, N: 13.87% (theor. C: 49.84%, H: 2.44%, N: 14.53%)

S15: methyl 2',4'-dinitro-[1,1'-biphenyl]-4-carboxylate (Entry 15-Table 1)

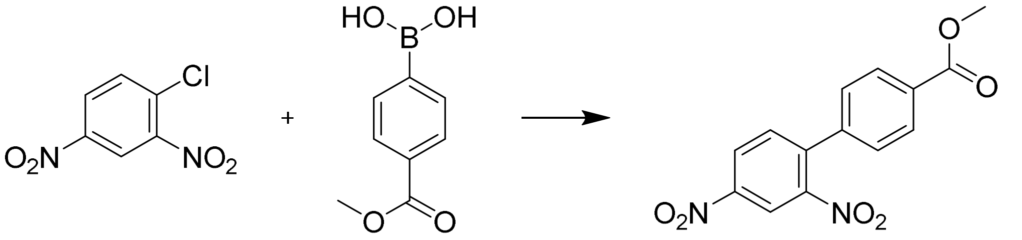

^{1}H NMR (400 MHz, Chloroform-*d*) δ 8.8 – 8.7 (d, *J* = 2.3 Hz), 8.5 – 8.5 (m), 8.1 – 8.1 (m), 7.7 – 7.7 (d, *J* = 8.4 Hz), 7.4 – 7.4 (m), 3.9 – 3.9 (m).

^{13}C NMR (125 MHz, Chlorform-*d*) δ 166.3, 148.9, 147.4, 141.5, 139.9, 133.3, 131.1, 130.3, 127.9, 126.9, 120.1, 76.9, 52.5.

Elemental analysis C: 55.44%, H: 3.46%, N: 9.02% (theor. C: 55.64%, H: 3.34%, N: 9.27%)

S16: methyl 2',4'-dinitro-[1,1'-biphenyl]-3-carboxylate (Entry 16-Table 1)

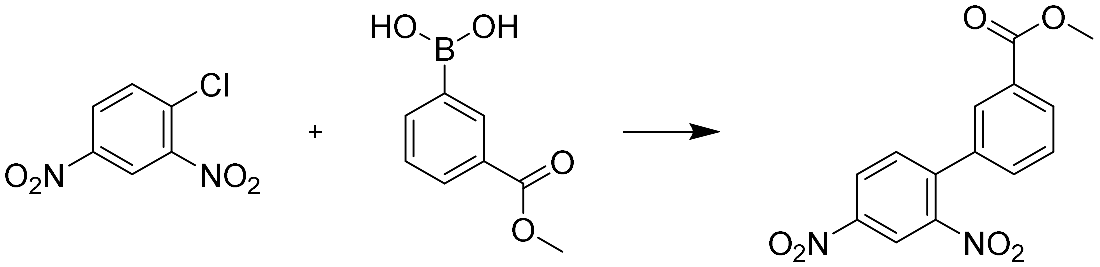

^{1}H NMR (400 MHz, Chloroform-*d*) δ 8.5 – 8.5 (dd, *J* = 8.5, 2.3 Hz, 1H), 8.2 – 8.1 (dt, *J* = 7.7, 1.5 Hz, 1H), 8.1 – 8.0 (t, *J* = 1.7 Hz, 1H), 7.7 – 7.7 (d, *J* = 8.5 Hz, 1H), 7.6 – 7.5 (td, *J* = 7.7, 0.6 Hz, 1H), 7.5 – 7.5 (ddd, *J* = 7.8, 1.9, 1.3 Hz, 1H), 4.0 – 3.9 (s, 6H).

^{13}C NMR NMR (101 MHz, Chloroform-*d*) δ 53.0, 77.2, 120.5, 127.2, 129.3, 129.7, 131.0, 131.6, 132.4, 133.8, 136.2, 141.8, 143.8.

Elemental analysis C: 54.89%, H: 3.24%, N: 8.9% (theor. C: 55.64%, H: 3.34%, N: 9.27%)

.S17: 2,3'-dinitro-1,1'-biphenyl (Entry 17-Table 1)

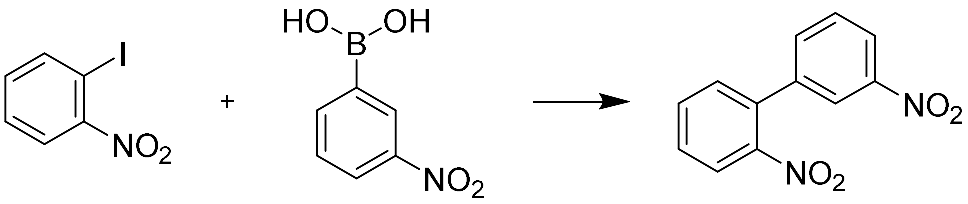

^{1}H NMR (400 MHz, Chloroform-*d*) δ 8.5 – 8.5 , 8.3 – 8.2 (dd, *J* = 5.5, 2.9 Hz), 8.2 – 8.2 , 8.0 – 8.0 (d, *J* = 8.5 Hz), 7.7 – 7.7 (m), 7.7 – 7.6 (d, *J* = 7.4 Hz), 7.5 – 7.4 (m).

^{13}C NMR (101 MHz, Chloroform-*d*) δ 77.2, 123.2, 123.2, 124.8, 129.6, 129.7, 132.1, 133.2, 134.2, 139.5, 148.4, 148.7.

S18: 2,2'-dinitro-1,1'-biphenyl (Entry 18-Table 1)

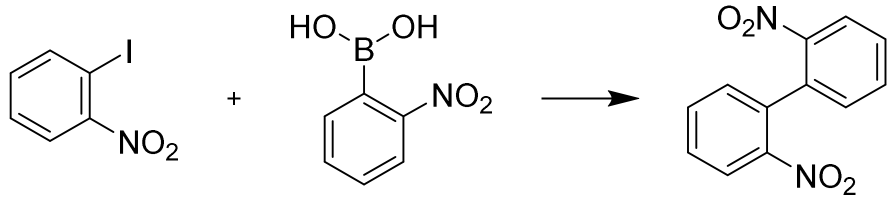

^{1}H NMR (400 MHz, Chloroform-*d*) δ 8.2 – 8.1 (d, *J* = 9.4 Hz), 7.7 – 7.6 (m), 7.6 – 7.5 (d, *J* = 7.9 Hz), 7.3 – 7.2 (m).

^{13}C NMR (101 MHz, Chloroform-*d*) δ 77.2, 124.9, 129.3, 131.0, 133.6, 134.3, 147.3.

S19: [N,N-dimethyl-3'-nitro-[1,1'-biphenyl]-4-amine

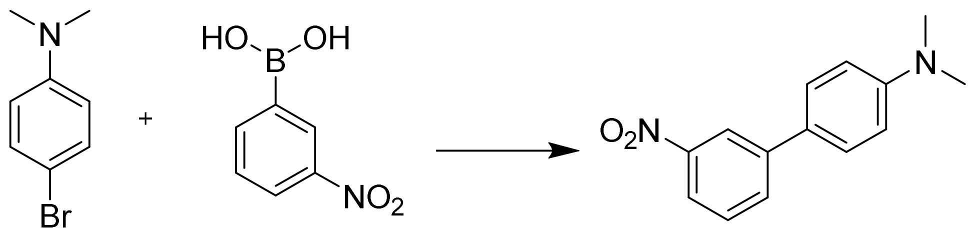

^{1}H NMR (400 MHz, Chloroform-*d*) δ 8.4 – 8.4 (t, *J* = 2.0 Hz), 8.1 – 8.0 (ddt, *J* = 8.2, 1.8, 0.9 Hz), 7.9 – 7.8 (ddd, *J* = 7.8, 1.8, 0.9 Hz), 7.6 – 7.5 (m), 6.8 – 6.8 (m), 3 (s).

^{13}C NMR (101 MHz, Chloroform-*d*) δ 40.4, 77.5, 112.7, 120.8, 126.1, 127.8, 129.6, 132.0, 143.0, 148.9, 150.8.

S16: dimethyl 2'-amino-2,2''-dinitro-[1,1':3',1''-terphenyl]-4,4''-dicarboxylate

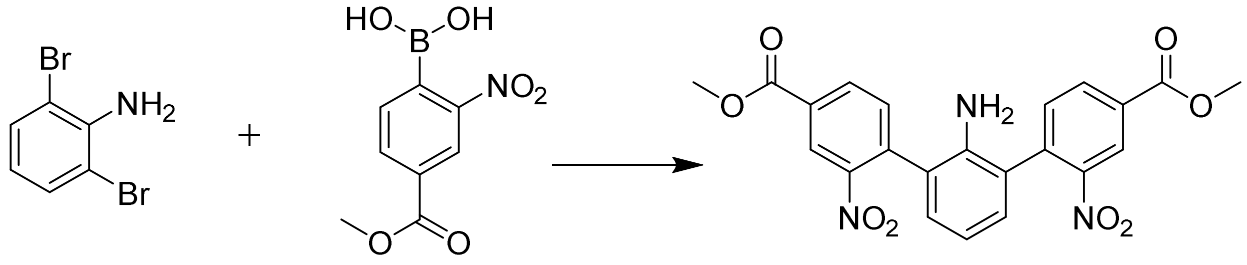

^{1}H NMR (400 MHz, Chloroform-*d*) δ 8.9 – 8.8 , , 8.8 – 8.7 , , 8.5 – 8.3 (dd, *J* = 19.6, 8.0 Hz), 8.2 – 8.2 (d, *J* = 8.8 Hz), 7.7 – 7.6 (t, *J* = 8.0 Hz), 7.2 – 7.2 (d, *J* = 8.7 Hz), 4.0 – 3.9 , , 4.0 – 3.8 , .

^{13}C NMR (101 MHz, Chloroform-*d*) δ 52.9, 120.3, 122.8, 124.7, 127.4, 127.5, 129.7, 131.9, 135.4, 138.0, 148.3, 158.2, 165.0.

NMR spectra of coupling products

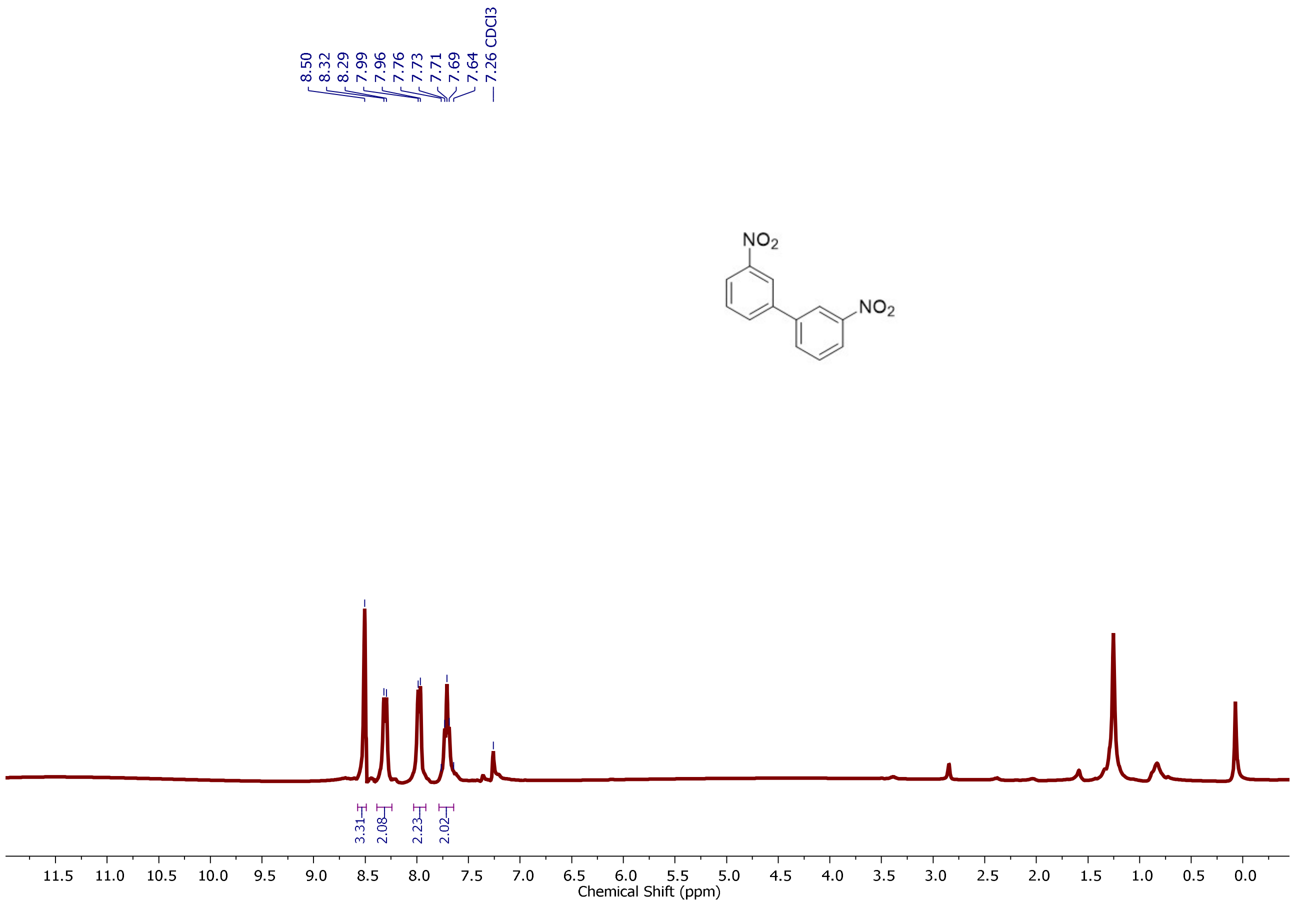

Figure S1a: ^{1}H NMR spectrum of S1:

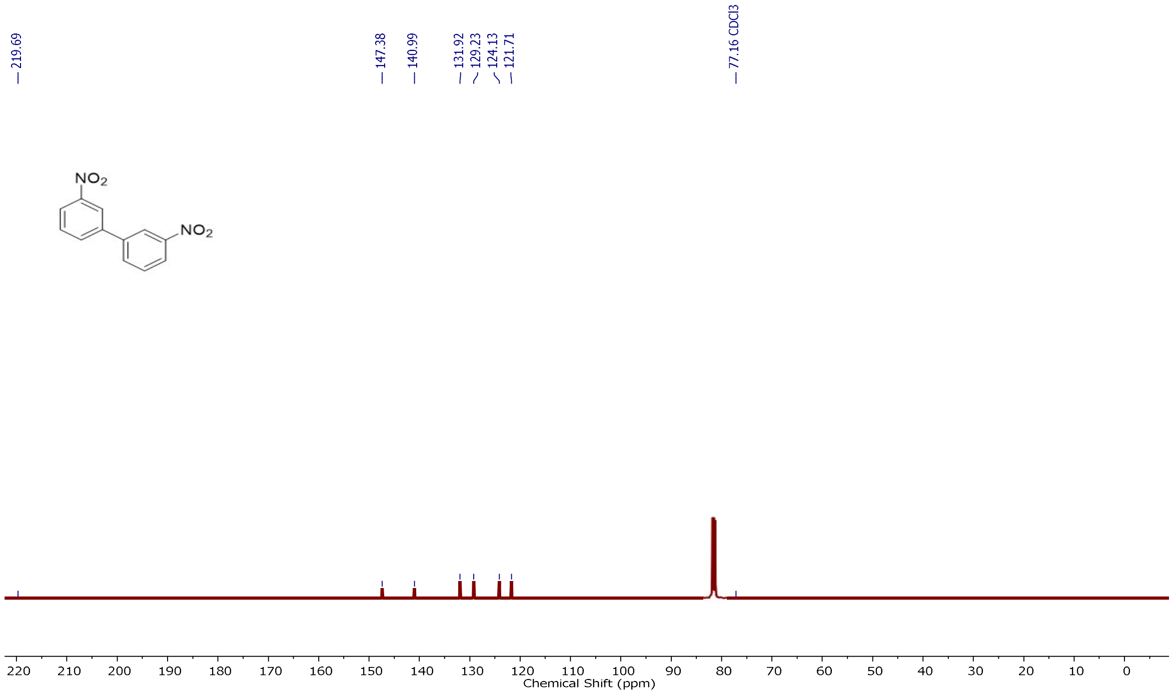

Figure S1b: ^{13}C spectrum of S1.

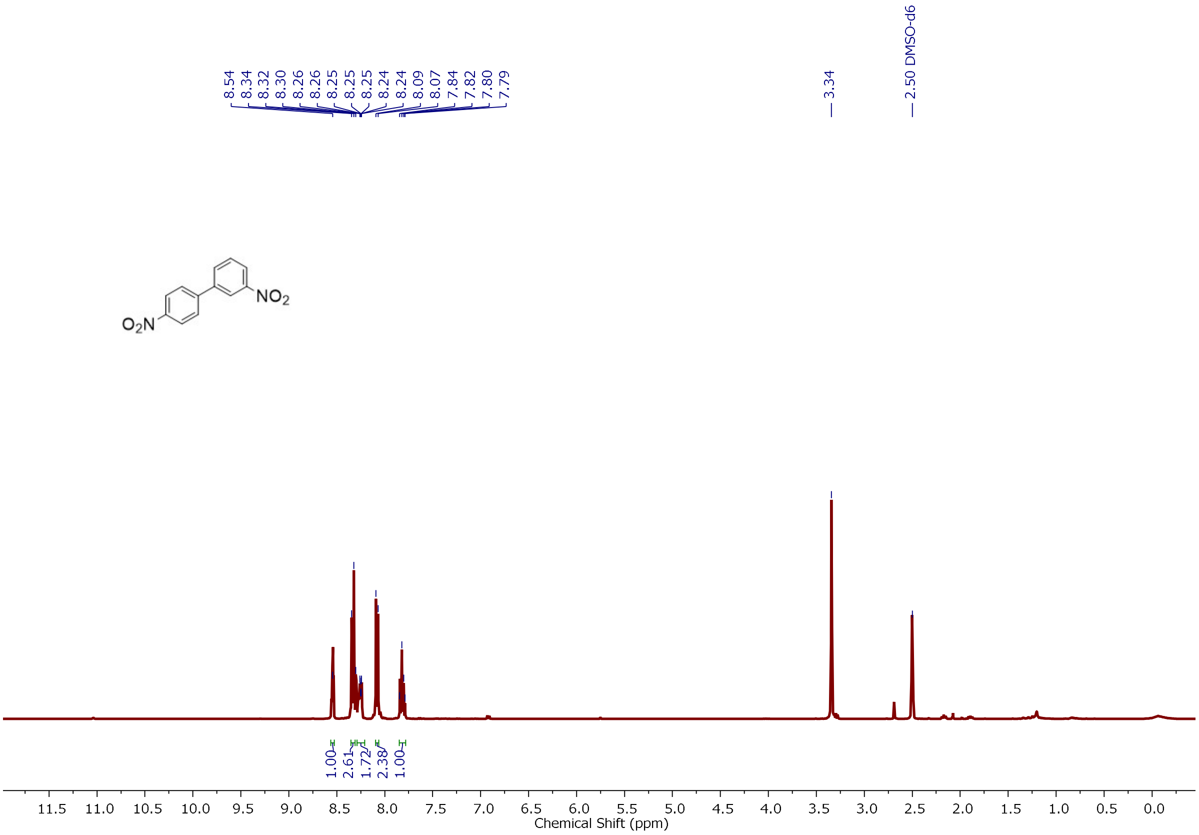

Figure S2a: ^{1}H NMR spectrum of S2.

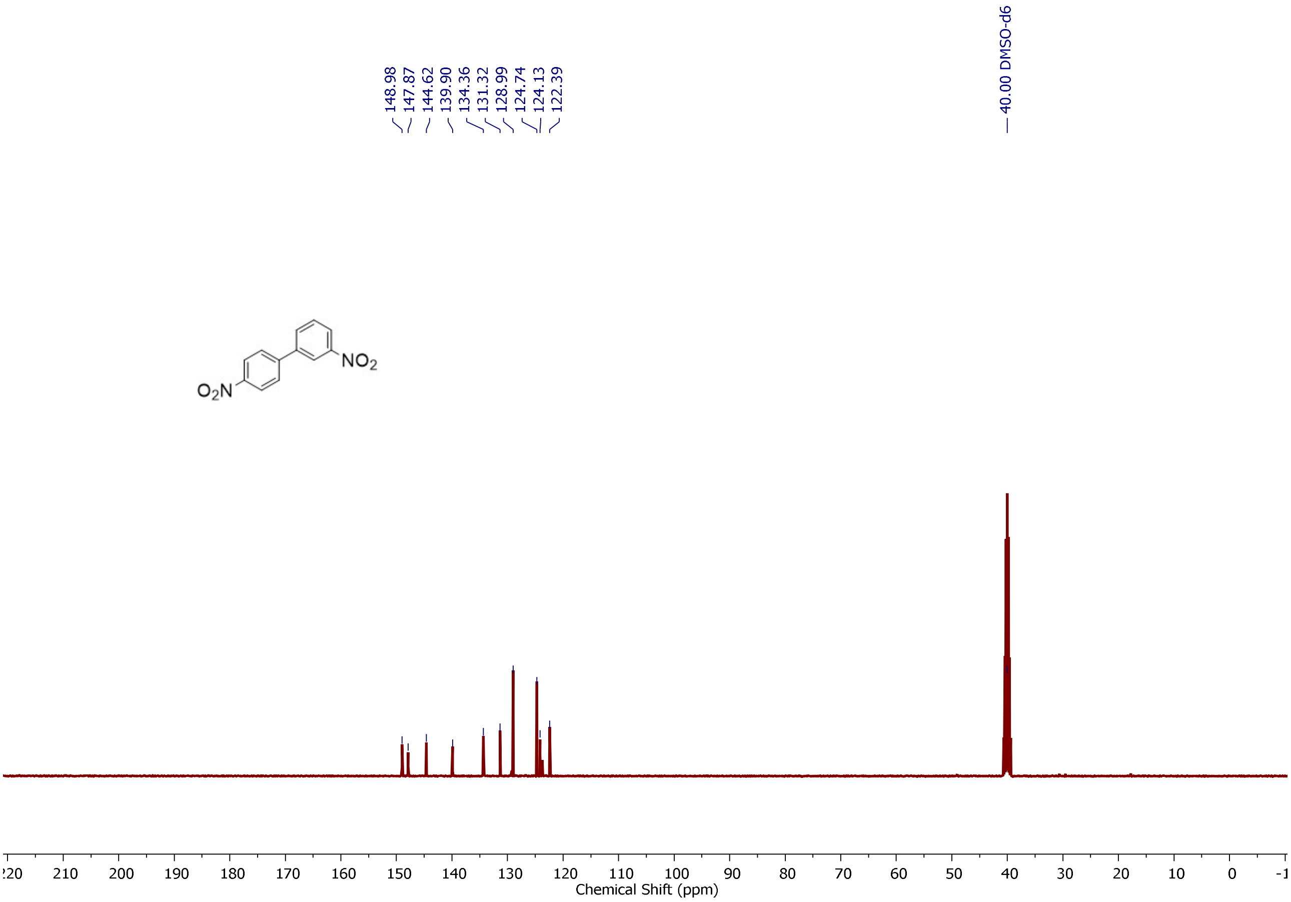

Figure S2b: ^{13}C NMR spectrum of S2.

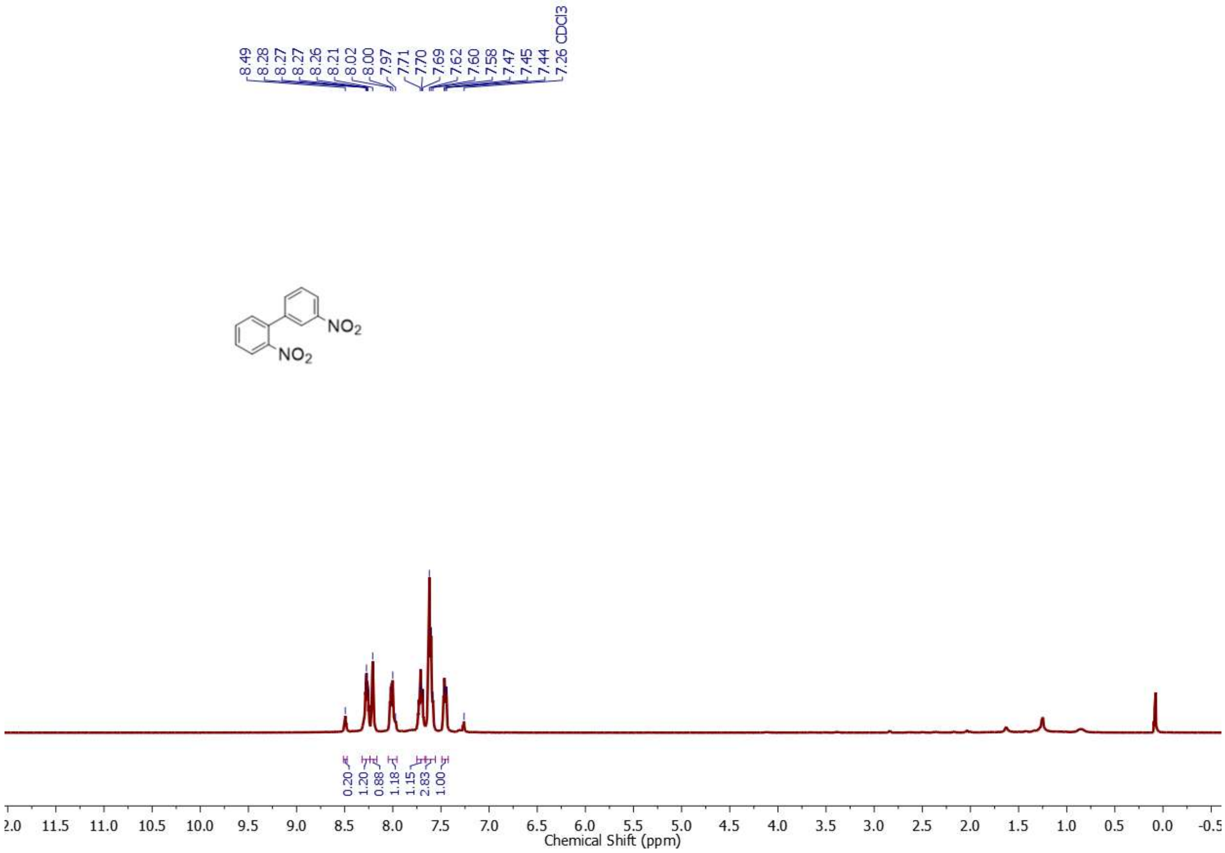

Figure S3a: ^{1}H NMR spectrum of S3.

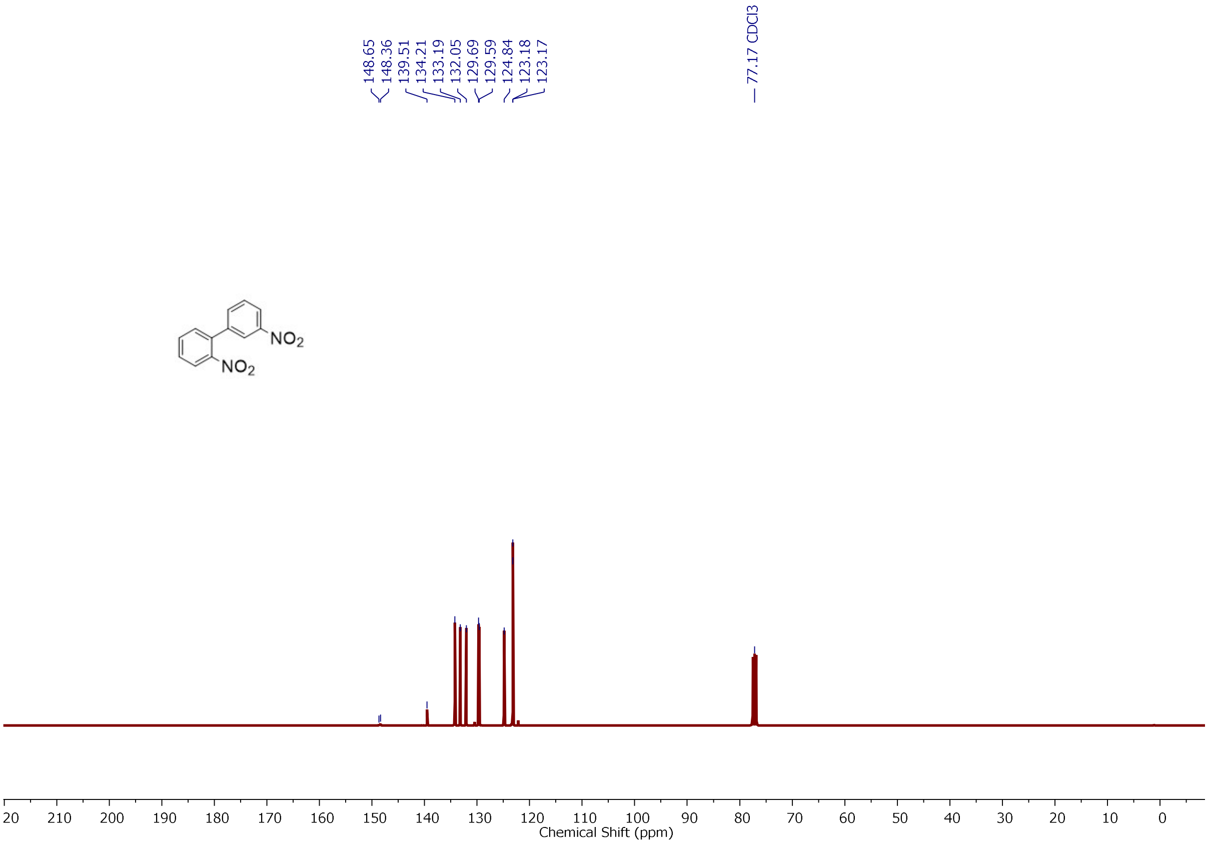

Figure S3b: ^{13}C NMR spectrum of S3.

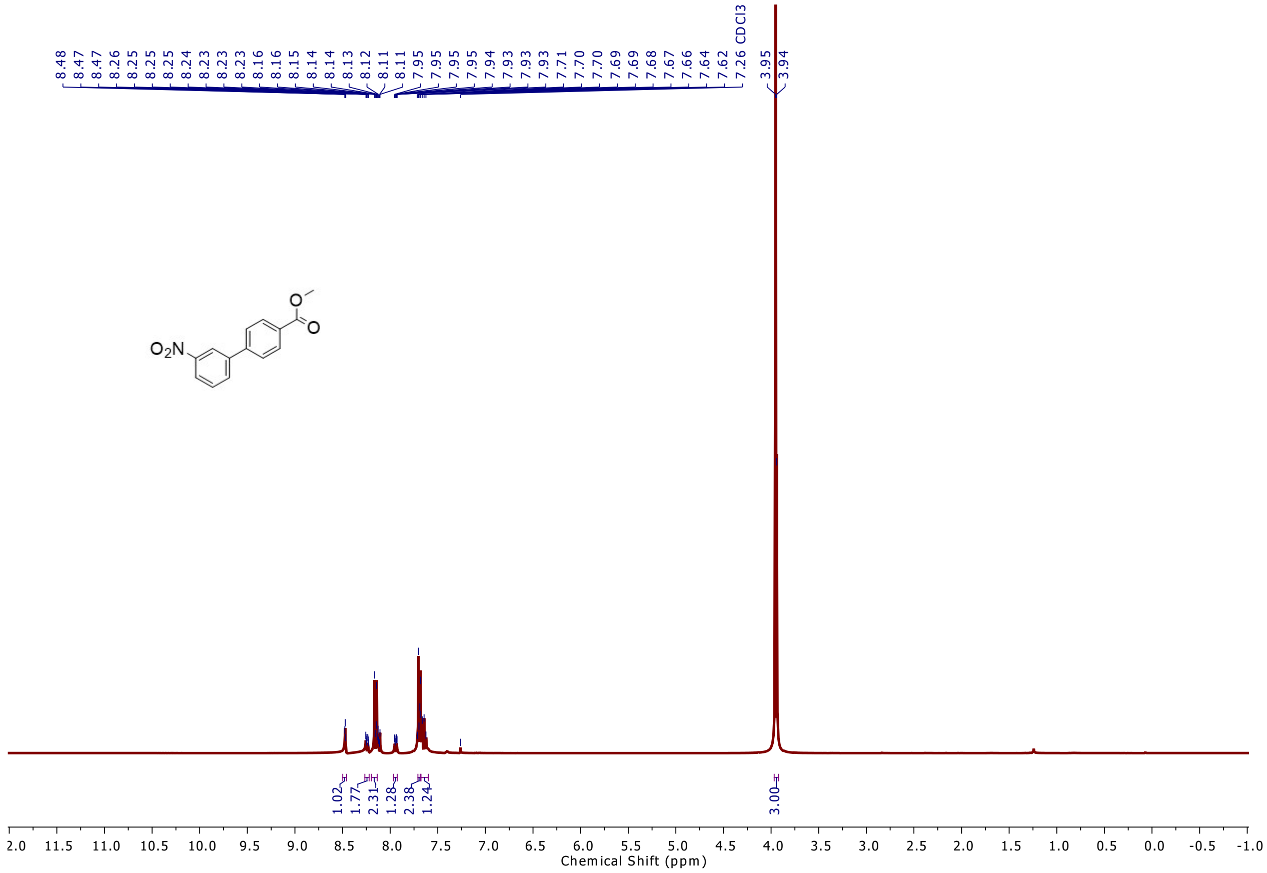

Figure S4a: ^{1}H NMR spectrum of S4.

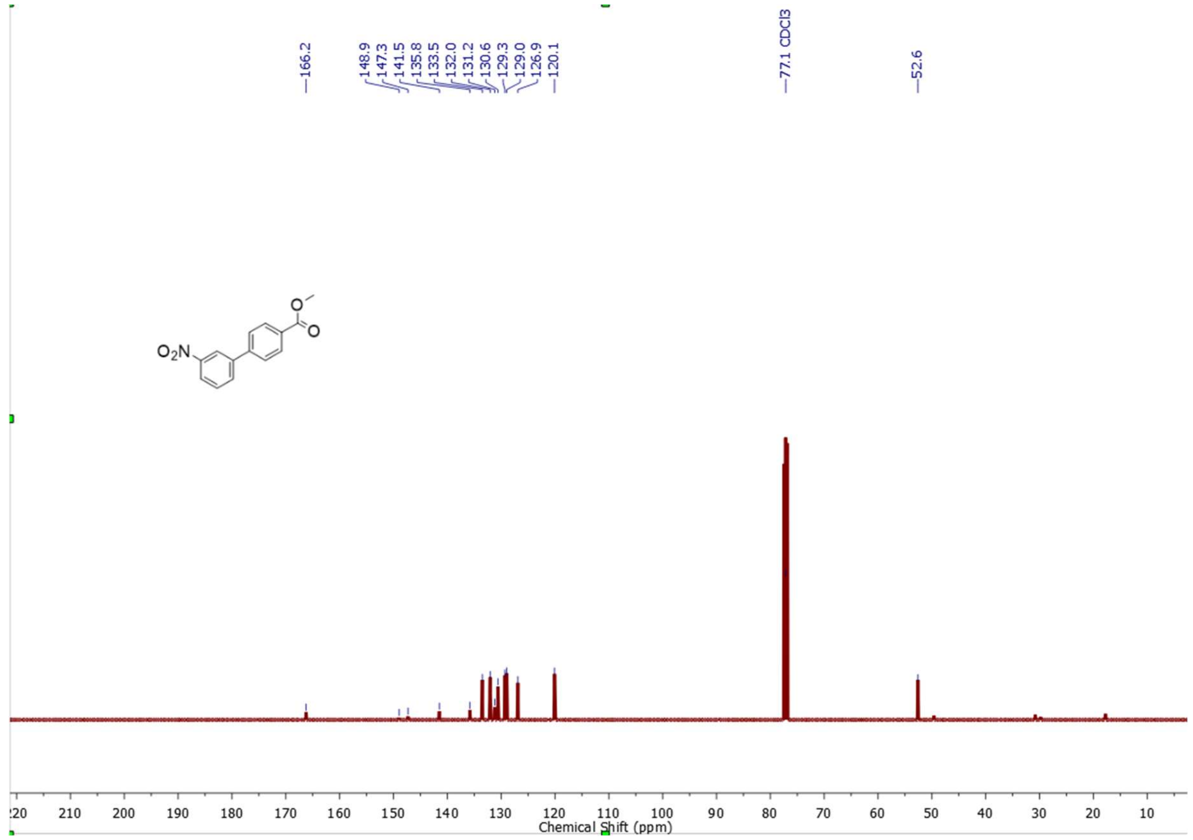

Figure S4b: ^{13}C NMR spectrum of S4.

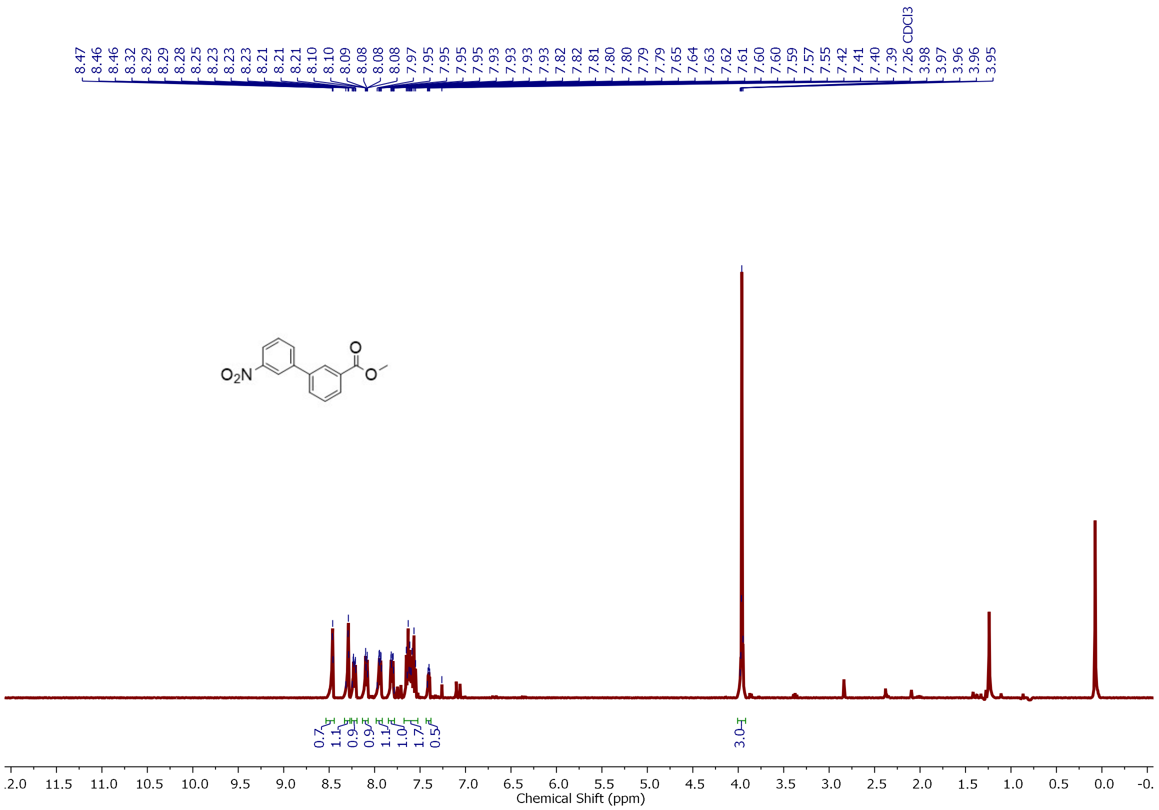

Figure S5a: ^{1}H NMR spectrum of S5: [nBu$_4$N]$_2$[methyldodecaborate].

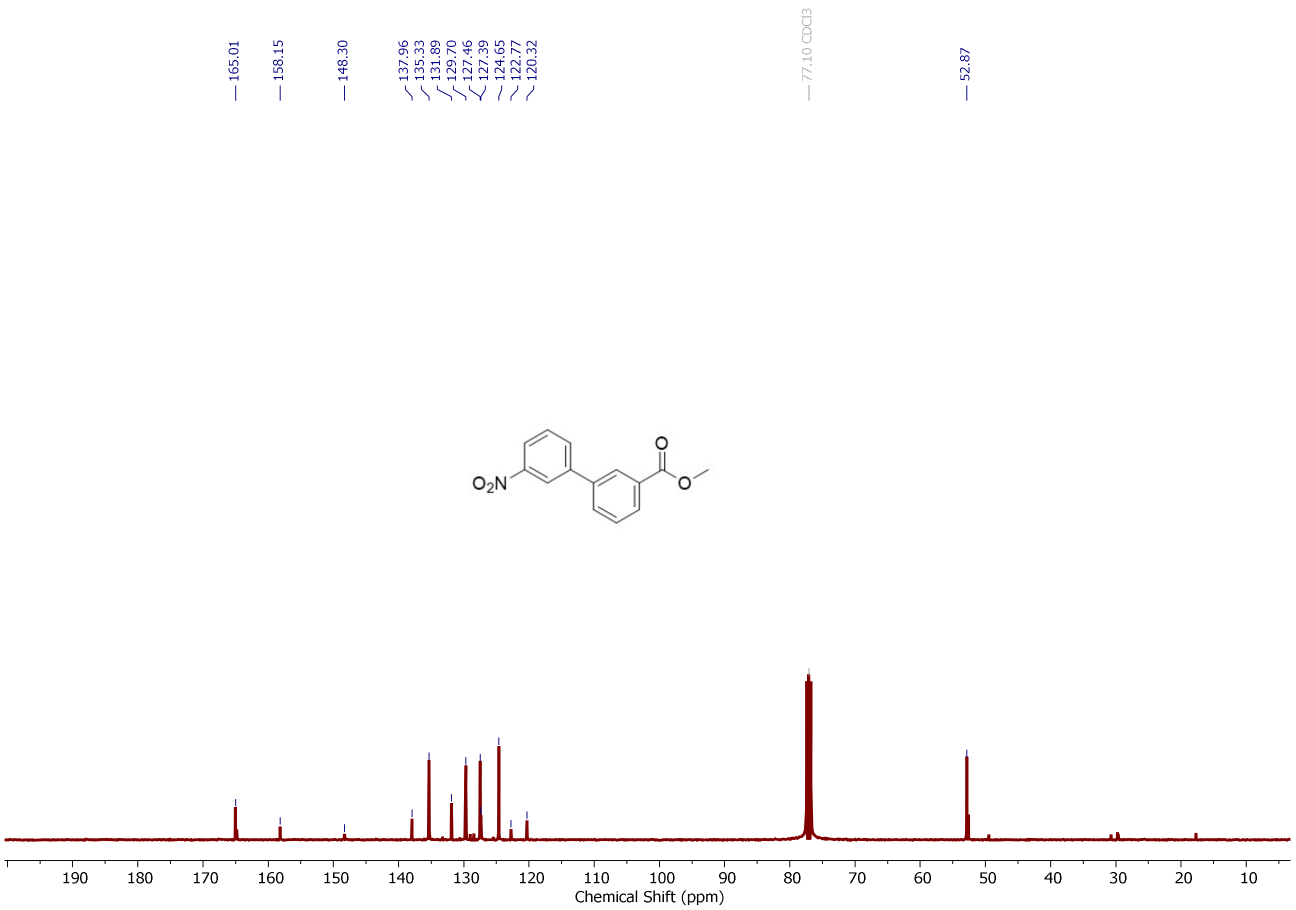

Figure S5b: ^{13}C NMR spectrum of S5.

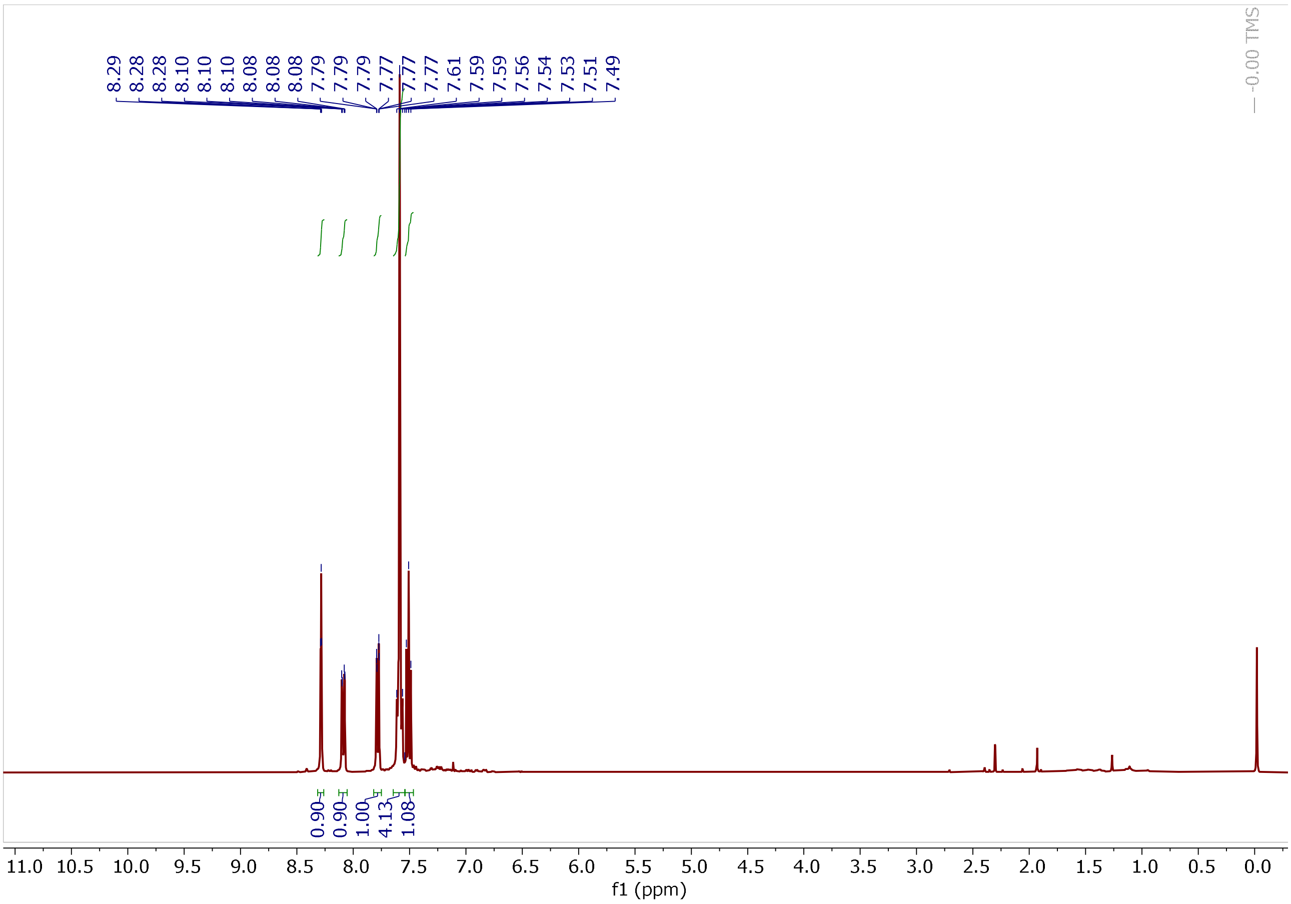

Figure S6a: ^{1}H NMR spectrum of S6.

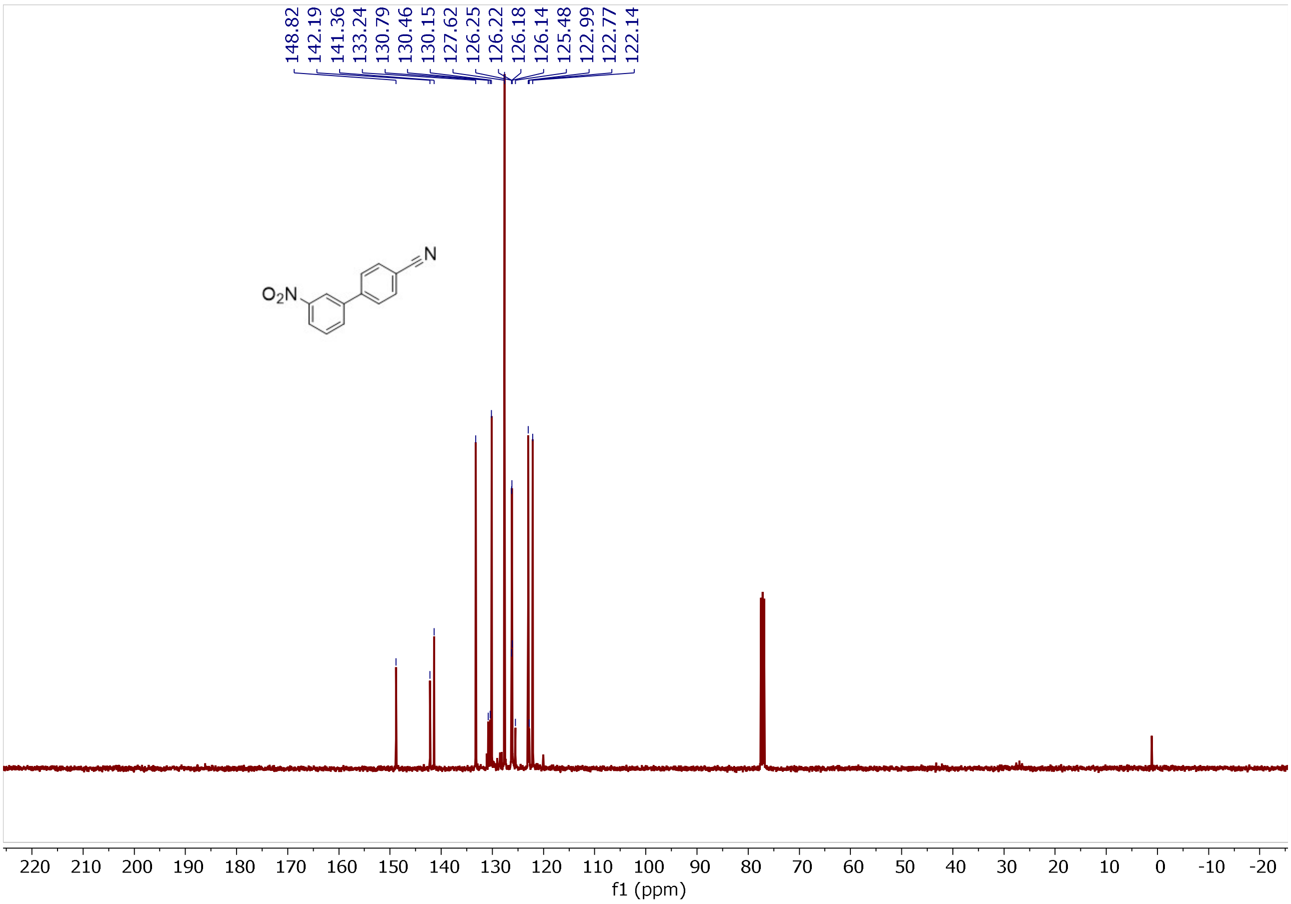

Figure S6a: ^{13}C NMR spectrum of S6.

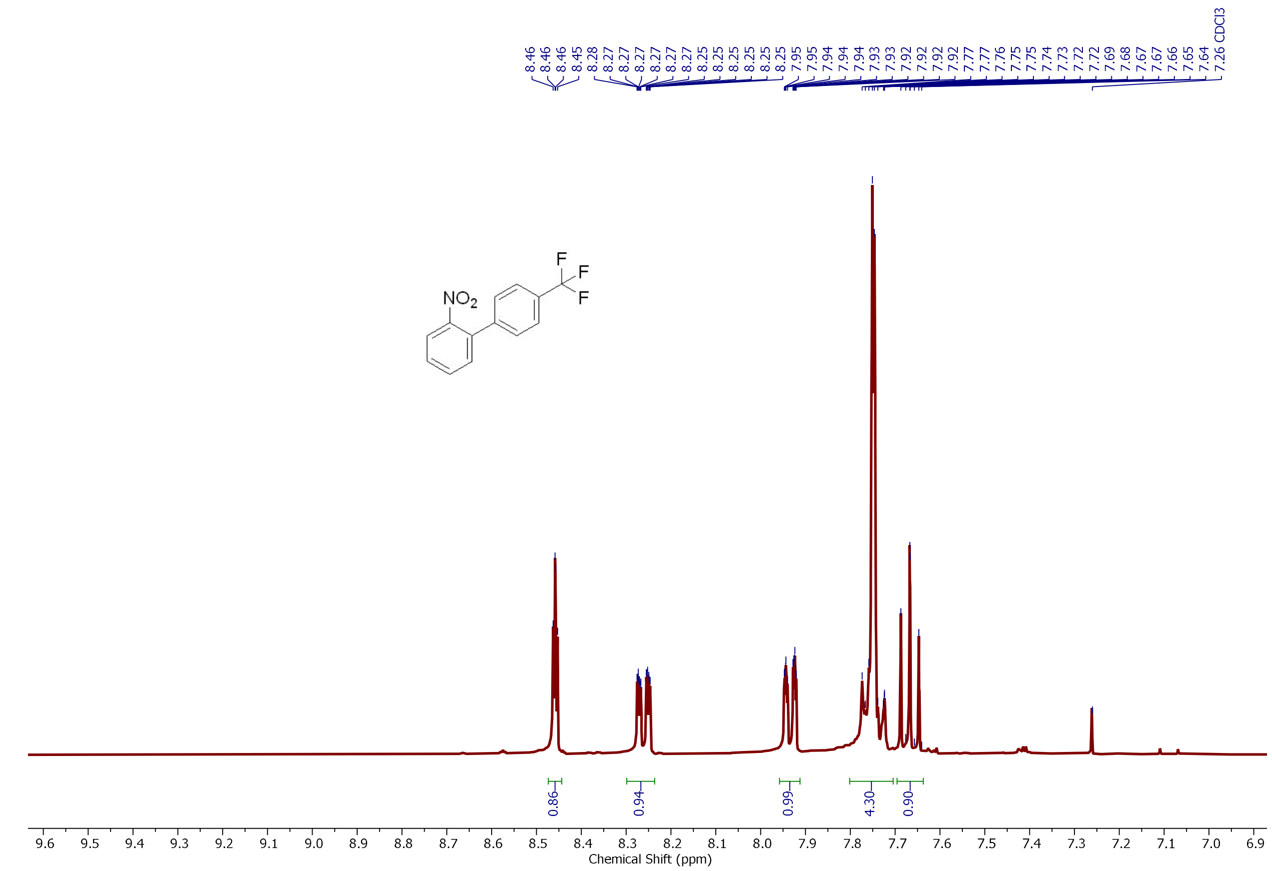

Figure S7a: ^{1}H NMR spectrum of S7.

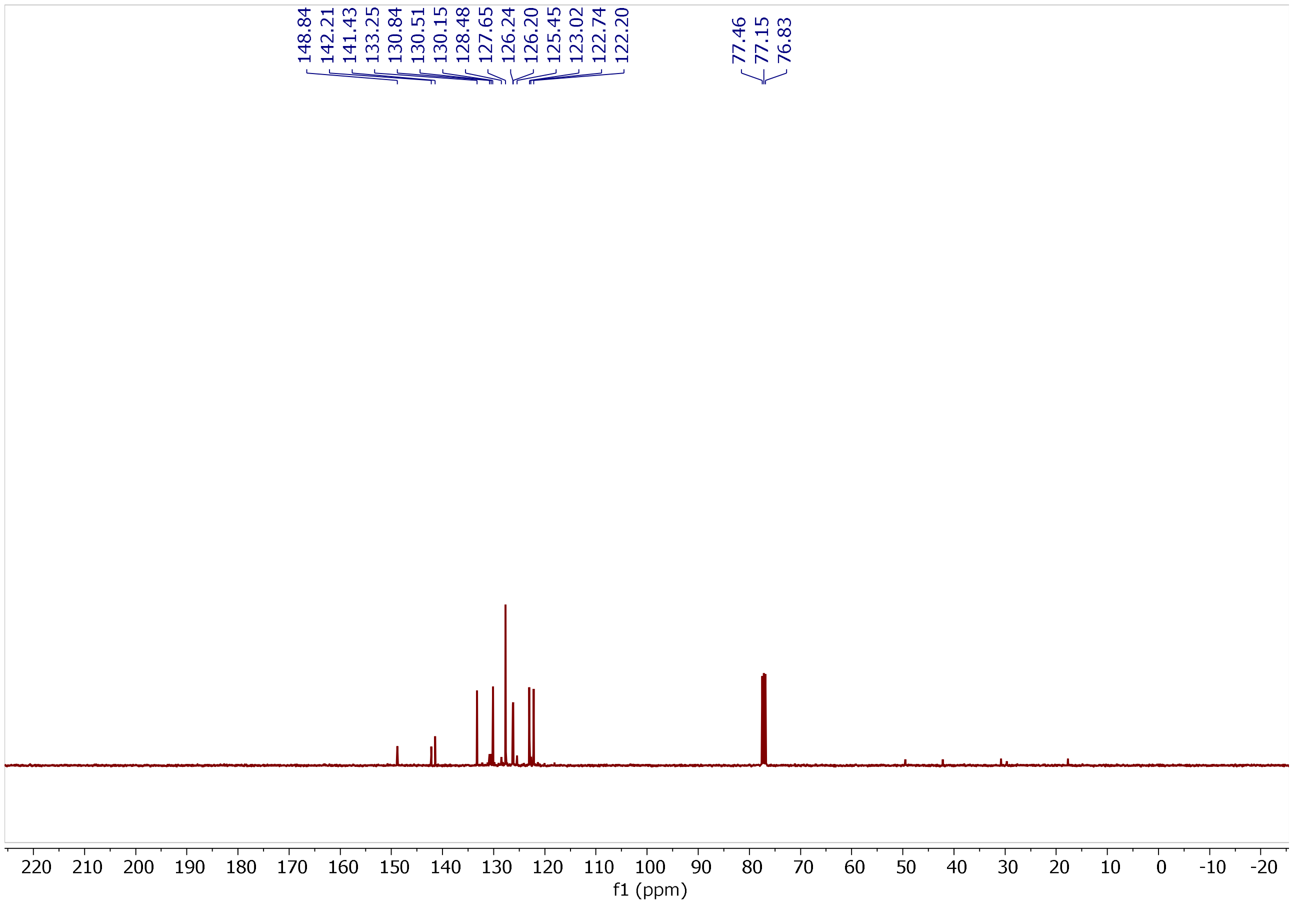

Figure S7a: ^{13}C NMR spectrum of S7.

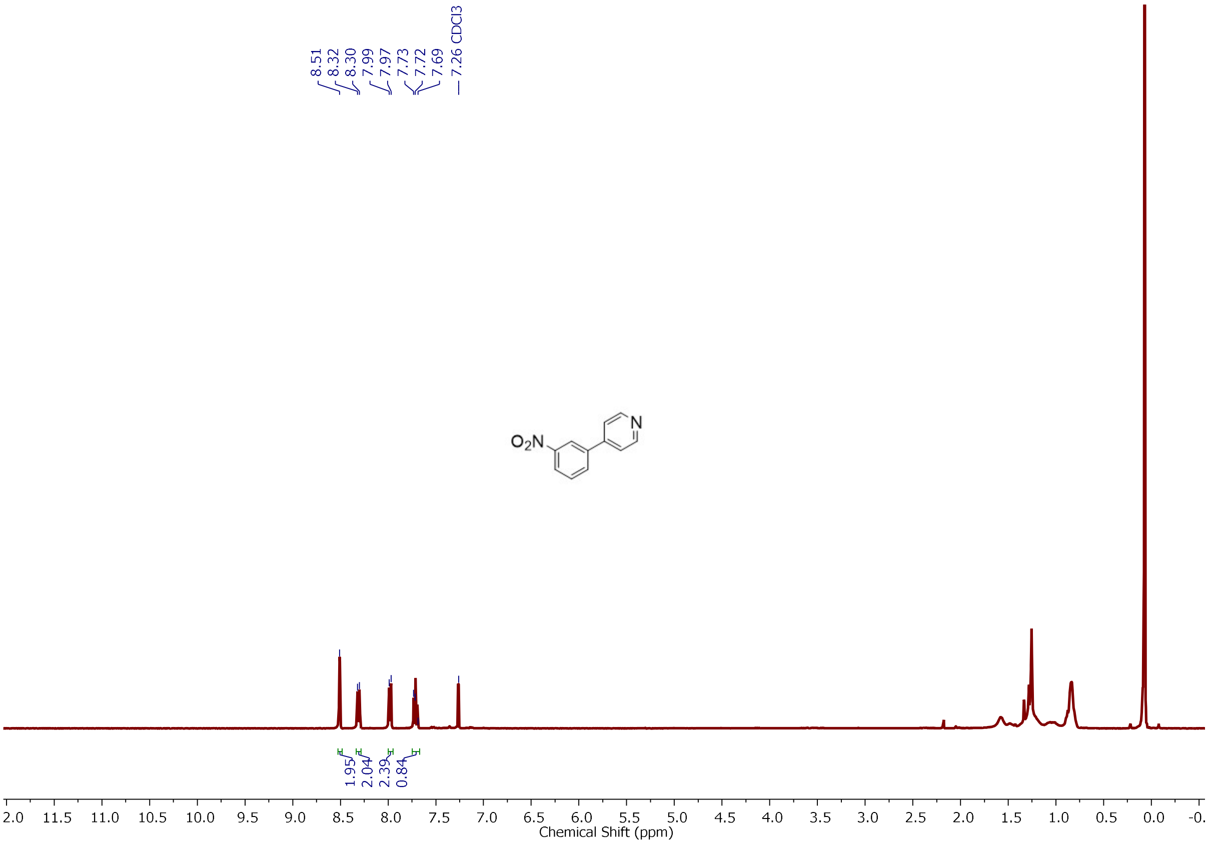

Figure S8a: ^{1}H NMR spectrum of S8.

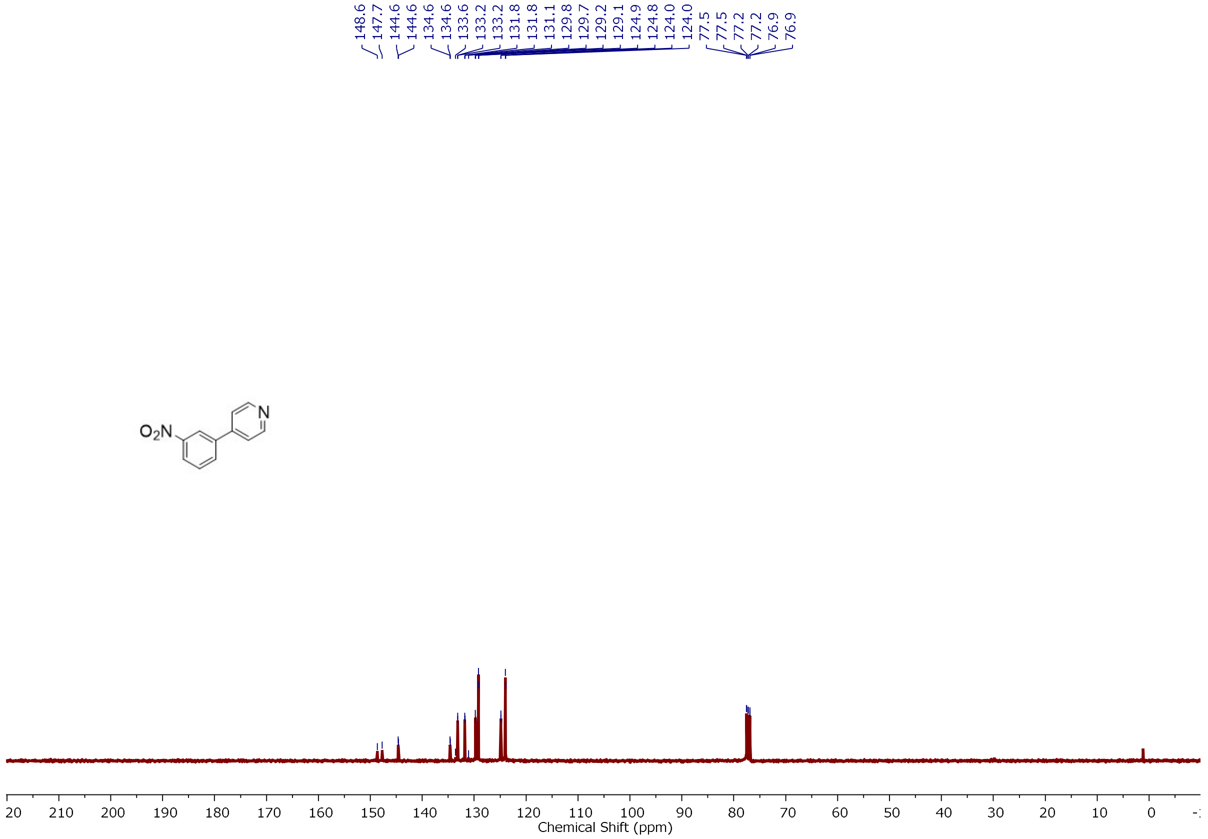

Figure S8b: ^{13}C NMR spectrum of S8.

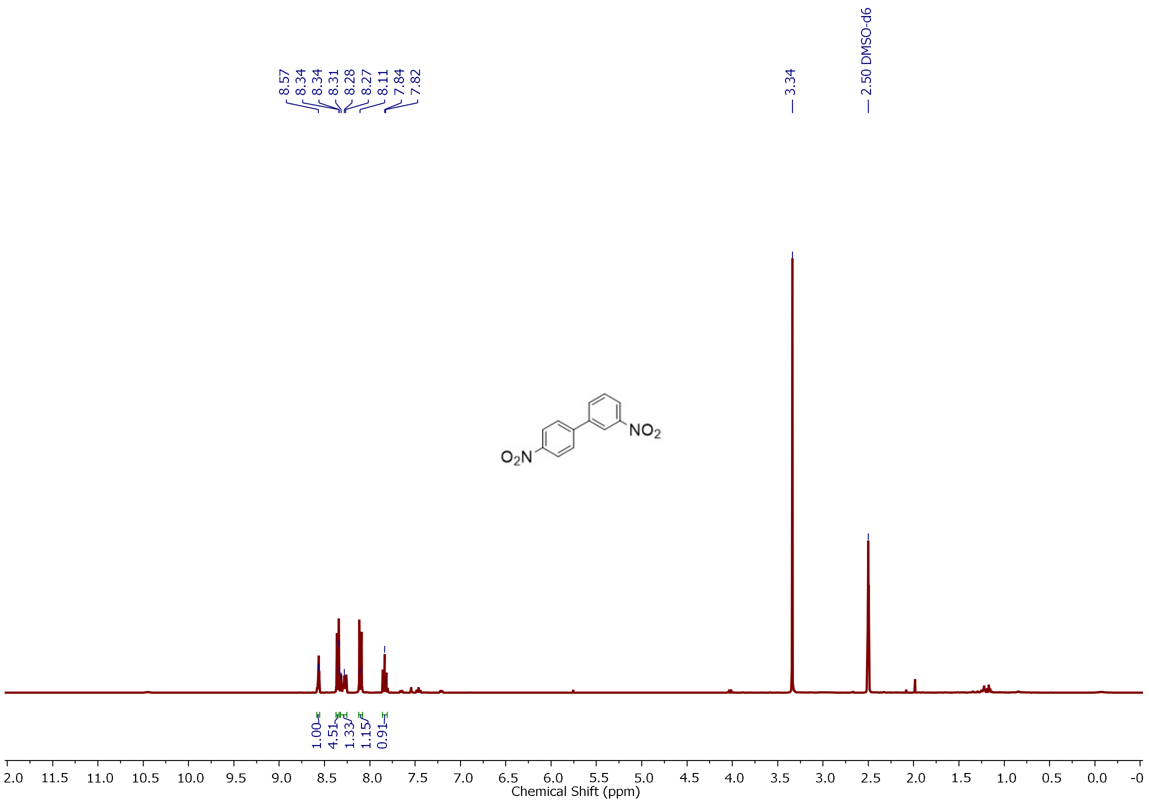

Figure S9a: ^{1}H NMR spectrum of S9.

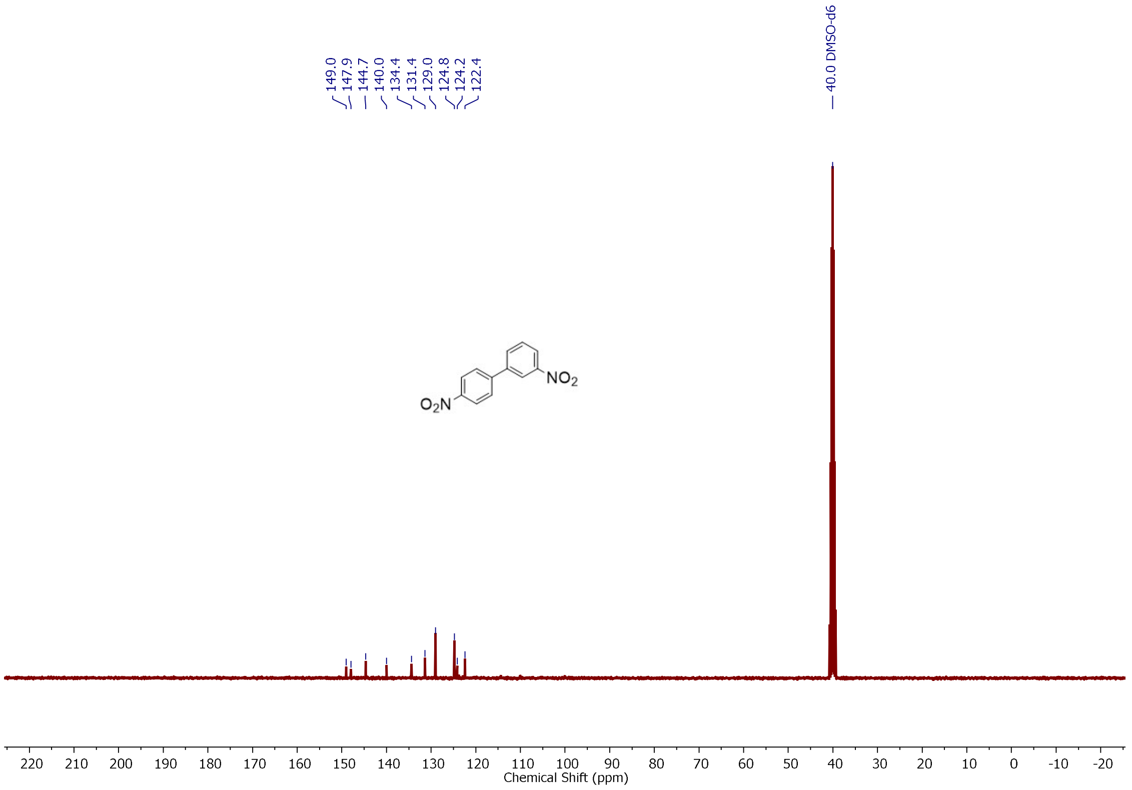

Figure S9b: ^{13}C NMR spectrum of S9.

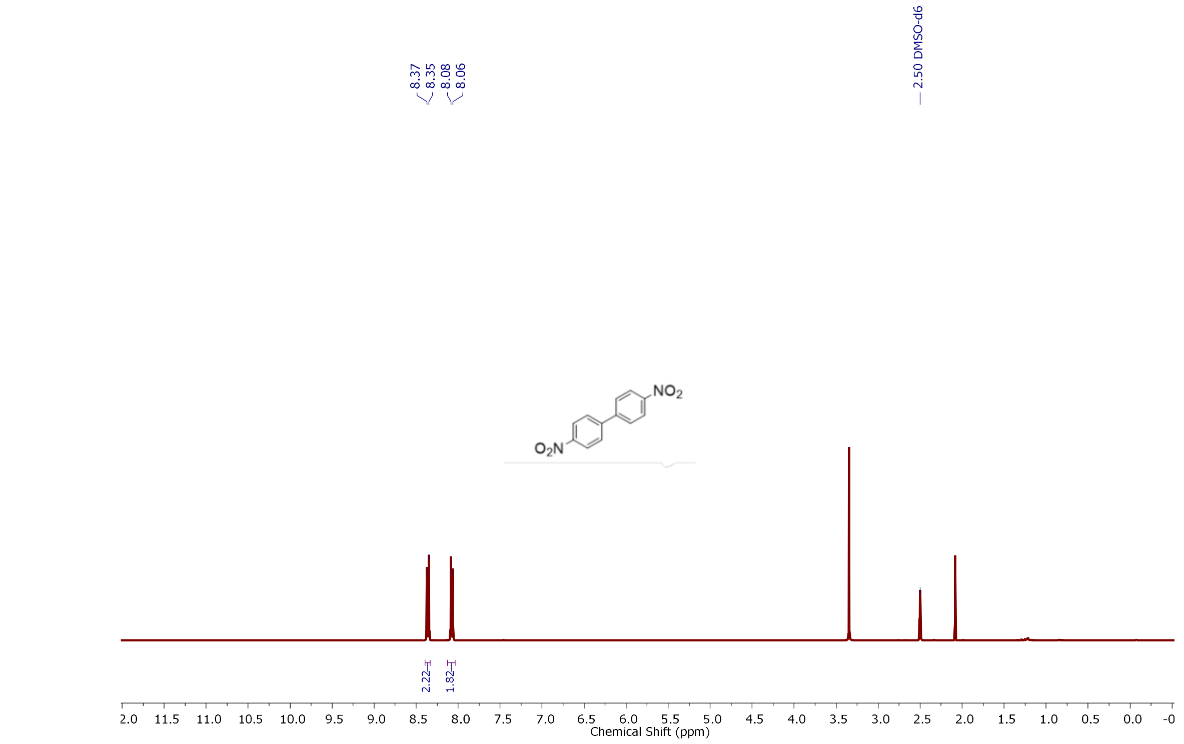

Figure S10a: ^{1}H NMR spectrum of S10.

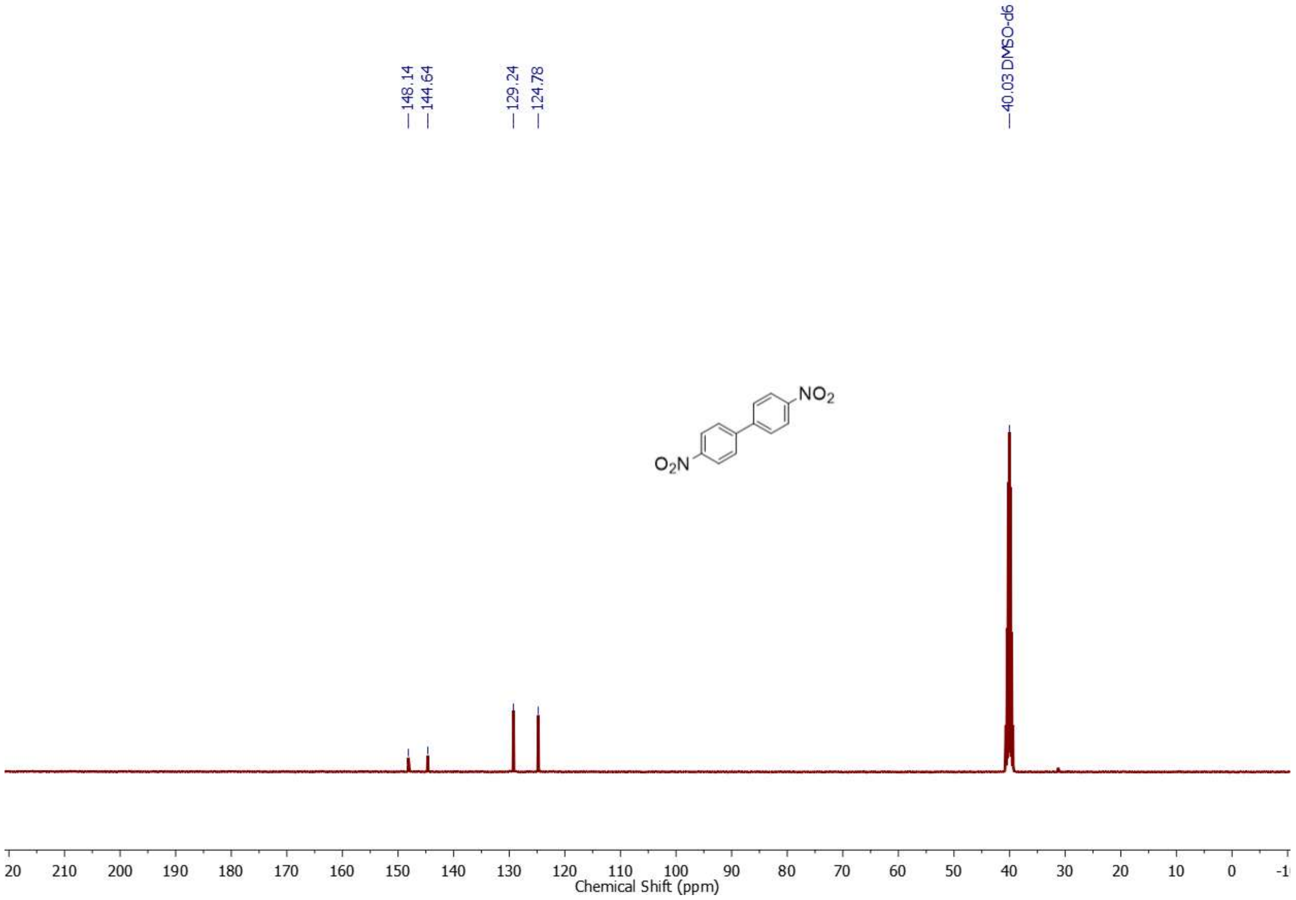

Figure S10b: ^{13}C NMR spectrum of S10.

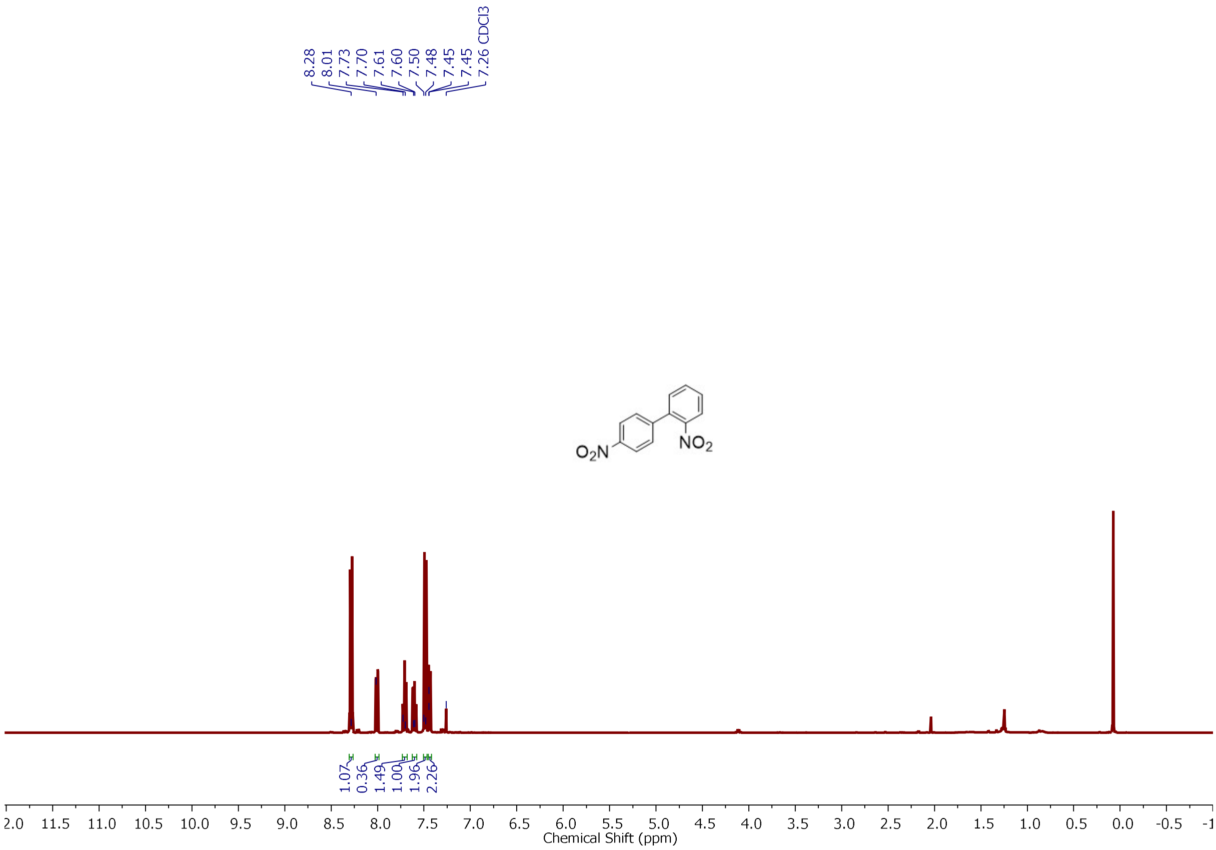

Figure S11a: ^{1}H NMR spectrum of S11.

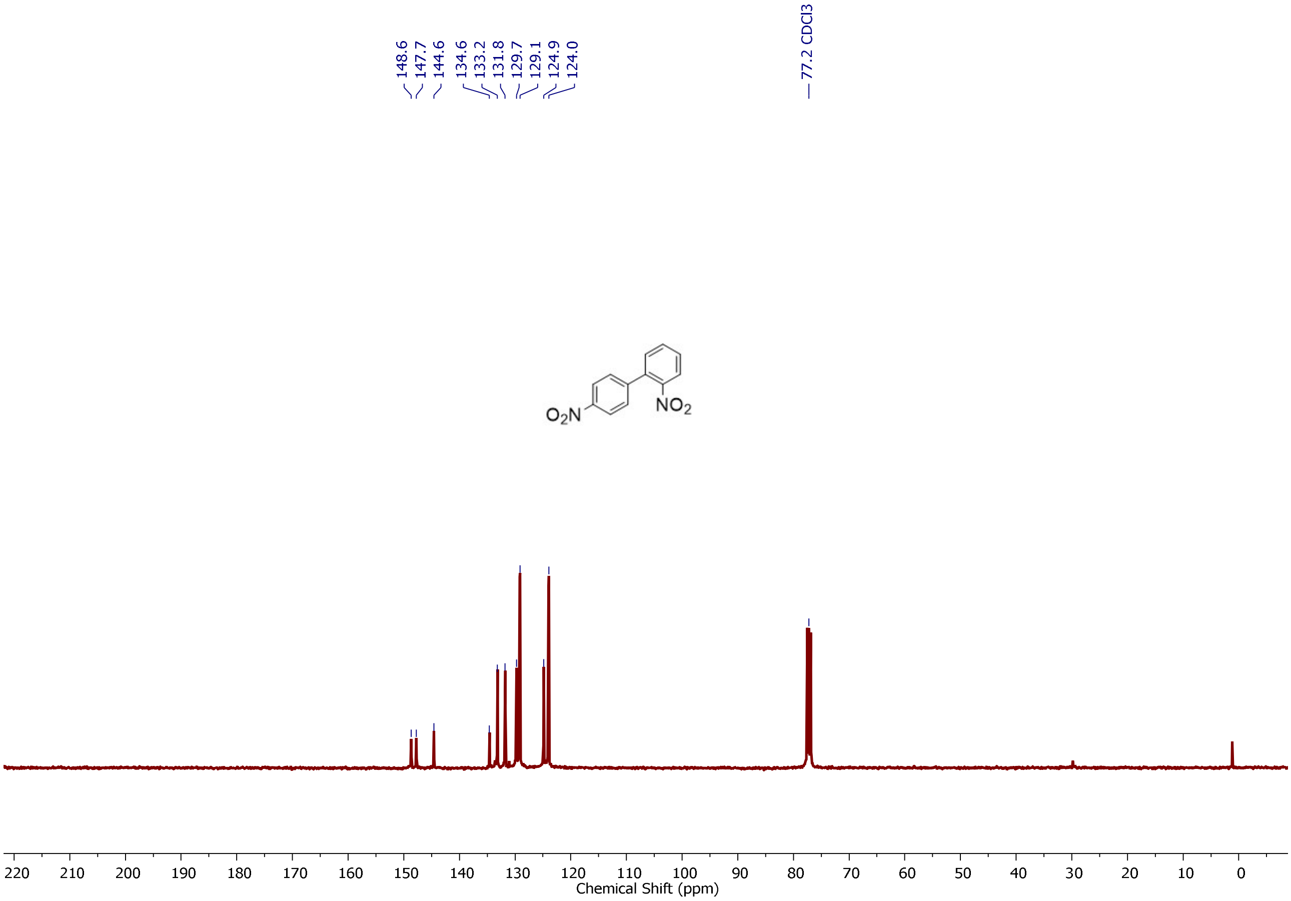

Figure S11b: ^{13}C NMR spectrum of S11.

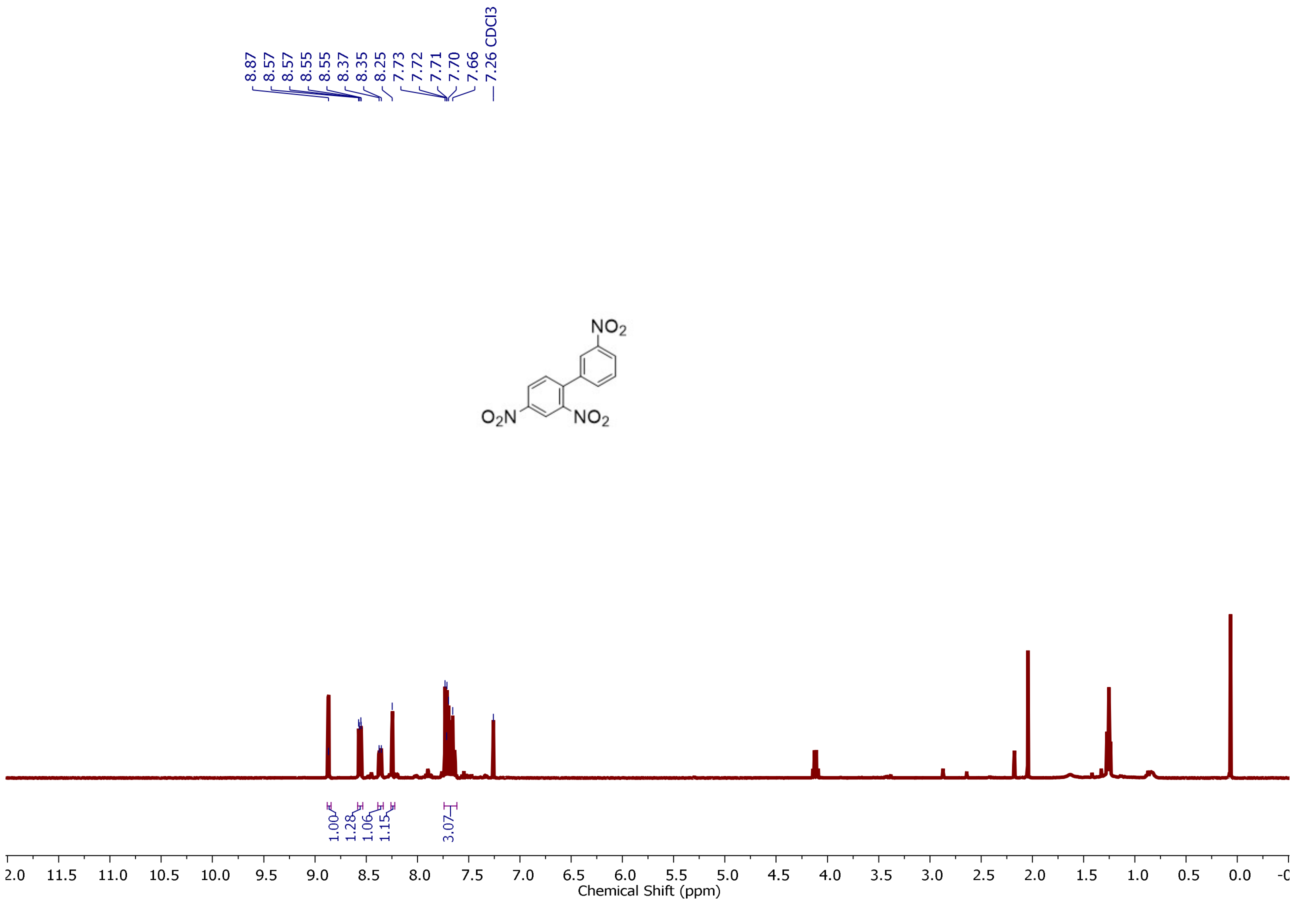

Figure S12a: ^{1}H NMR spectrum of S12.

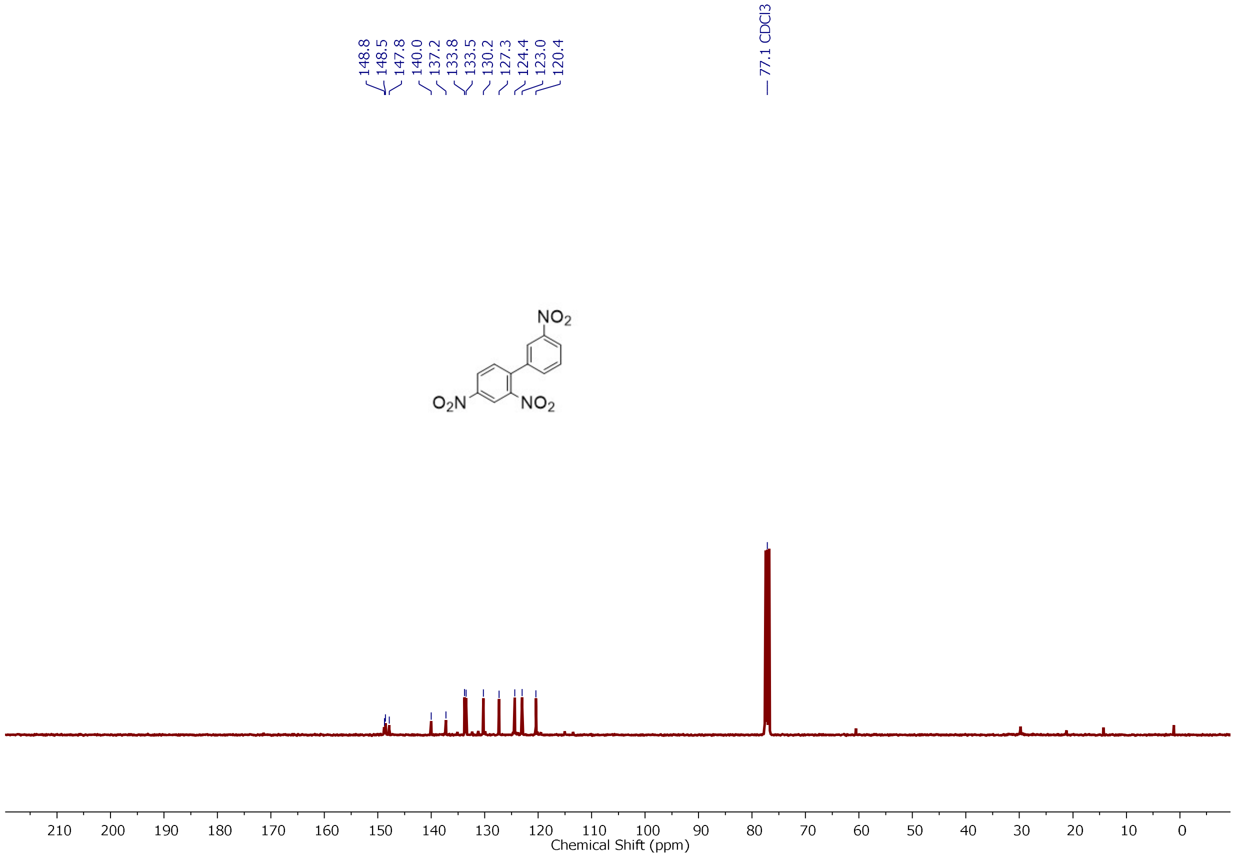

Figure S12b: ^{13}C NMR spectrum of S12.

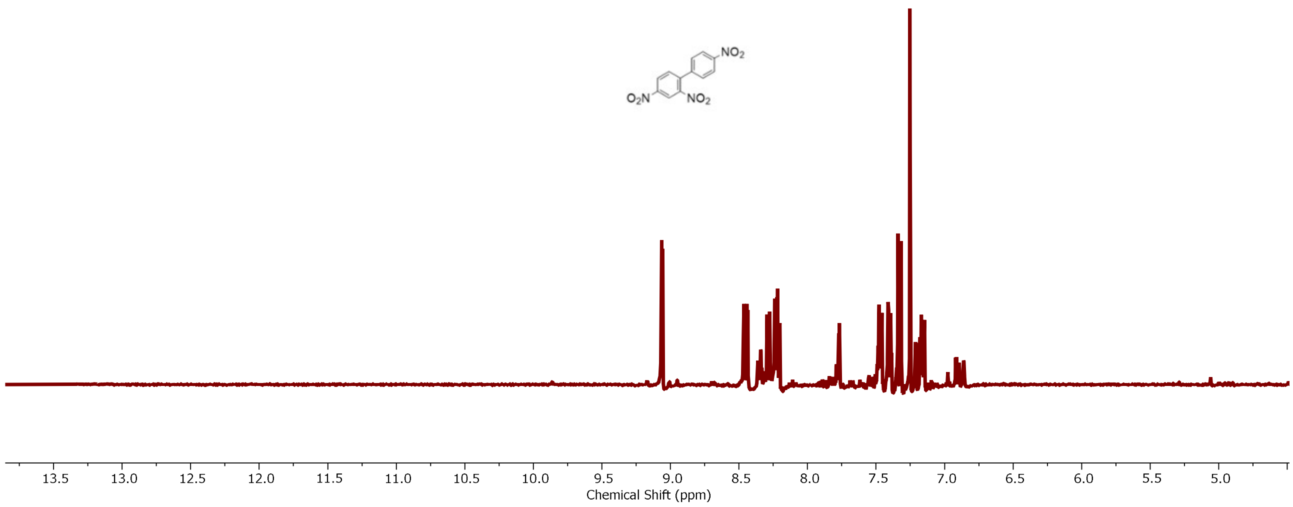

Figure S13a: ^{1}H NMR spectrum of S13, obtained as mixture with the Ullmann product.

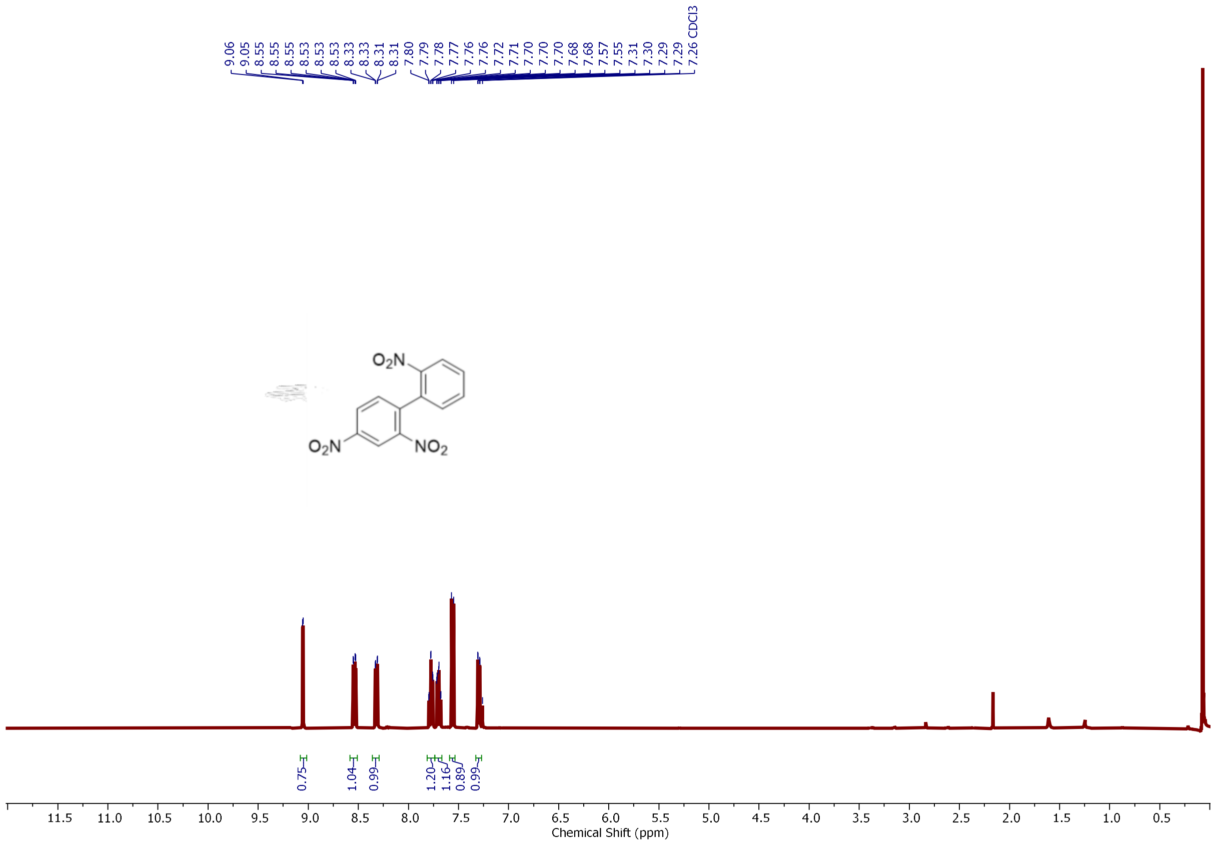

Figure S14a: ^{1}H NMR spectrum of S14.

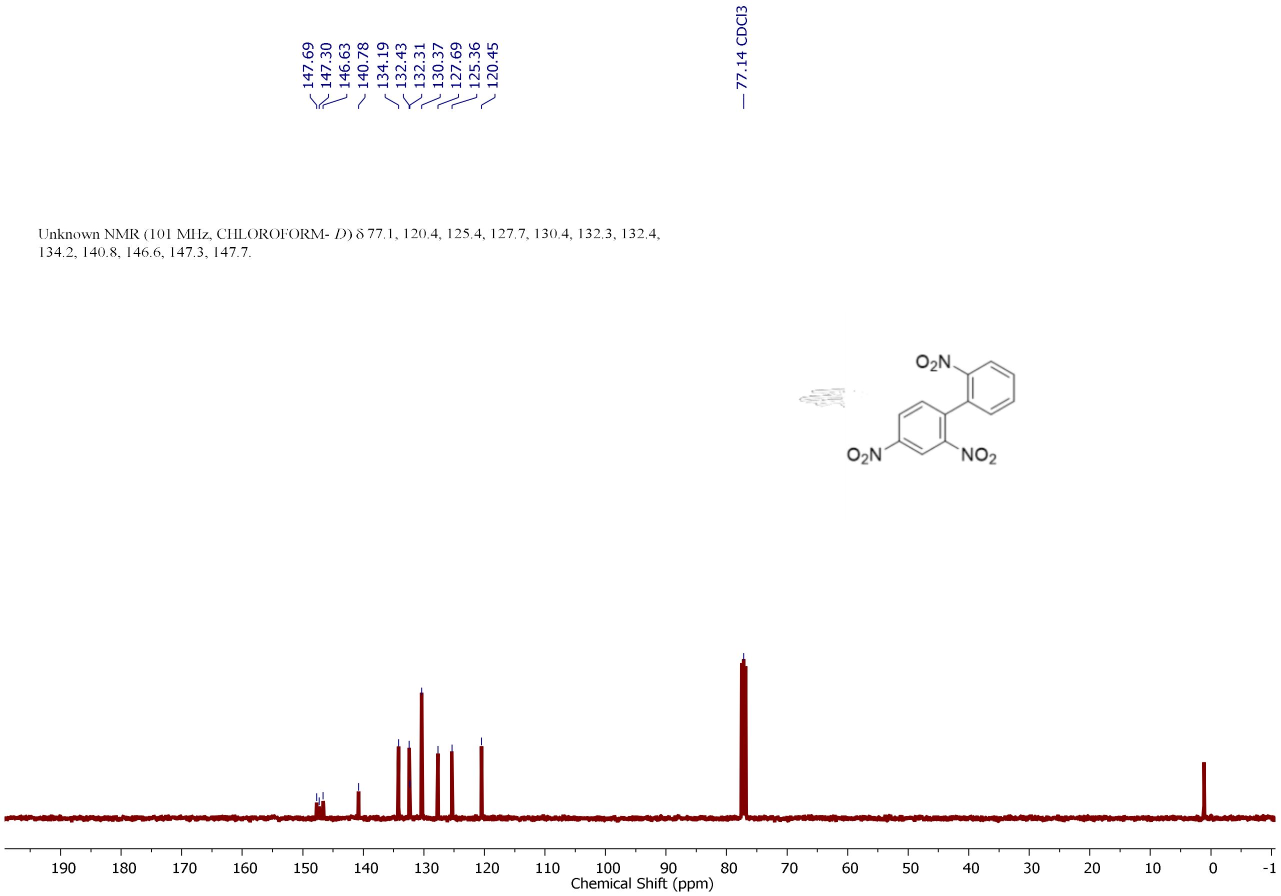

Figure S14b: ^{13}C NMR spectrum of S14.

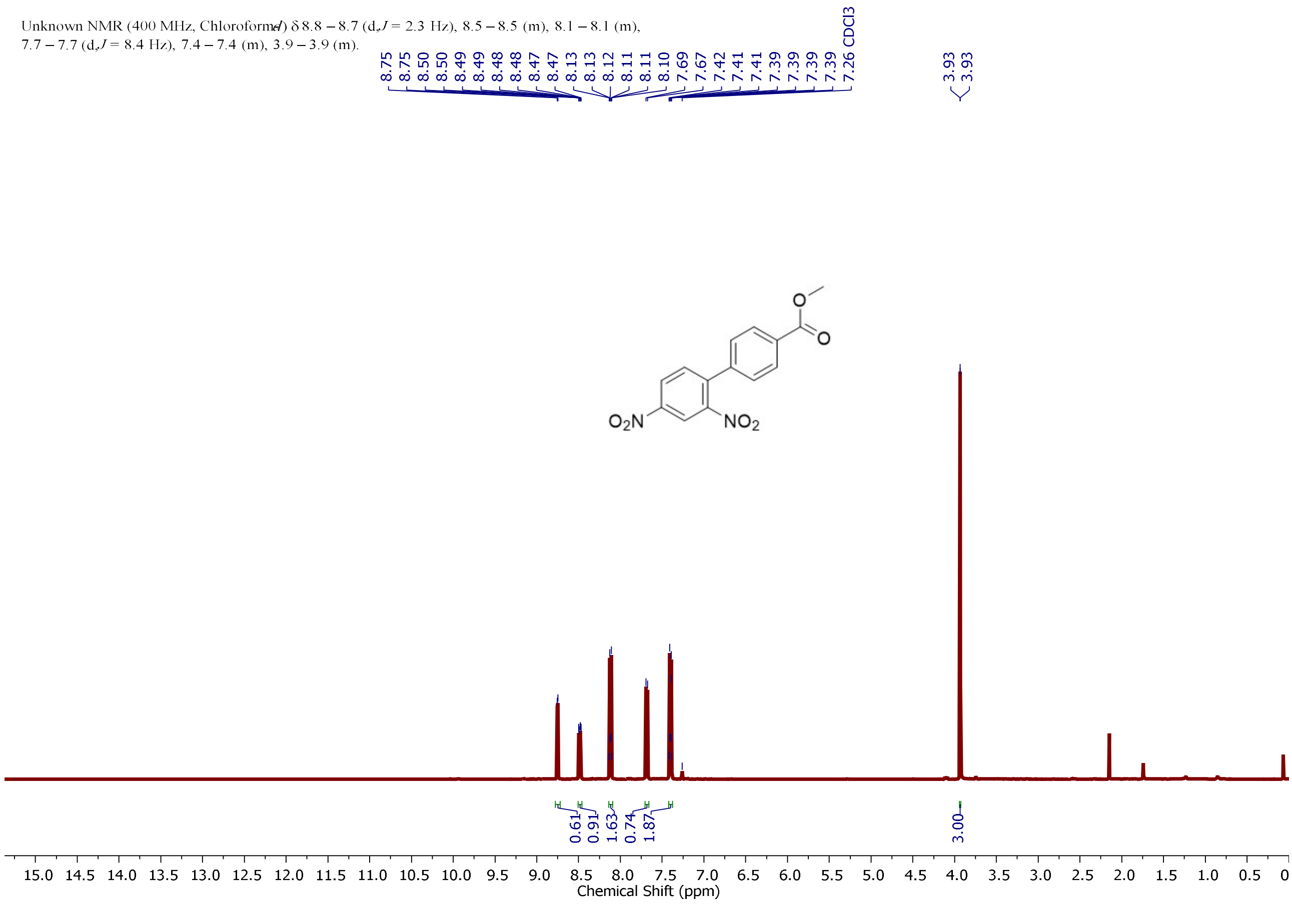

Figure S15a: ^{1}H NMR spectrum of S15.

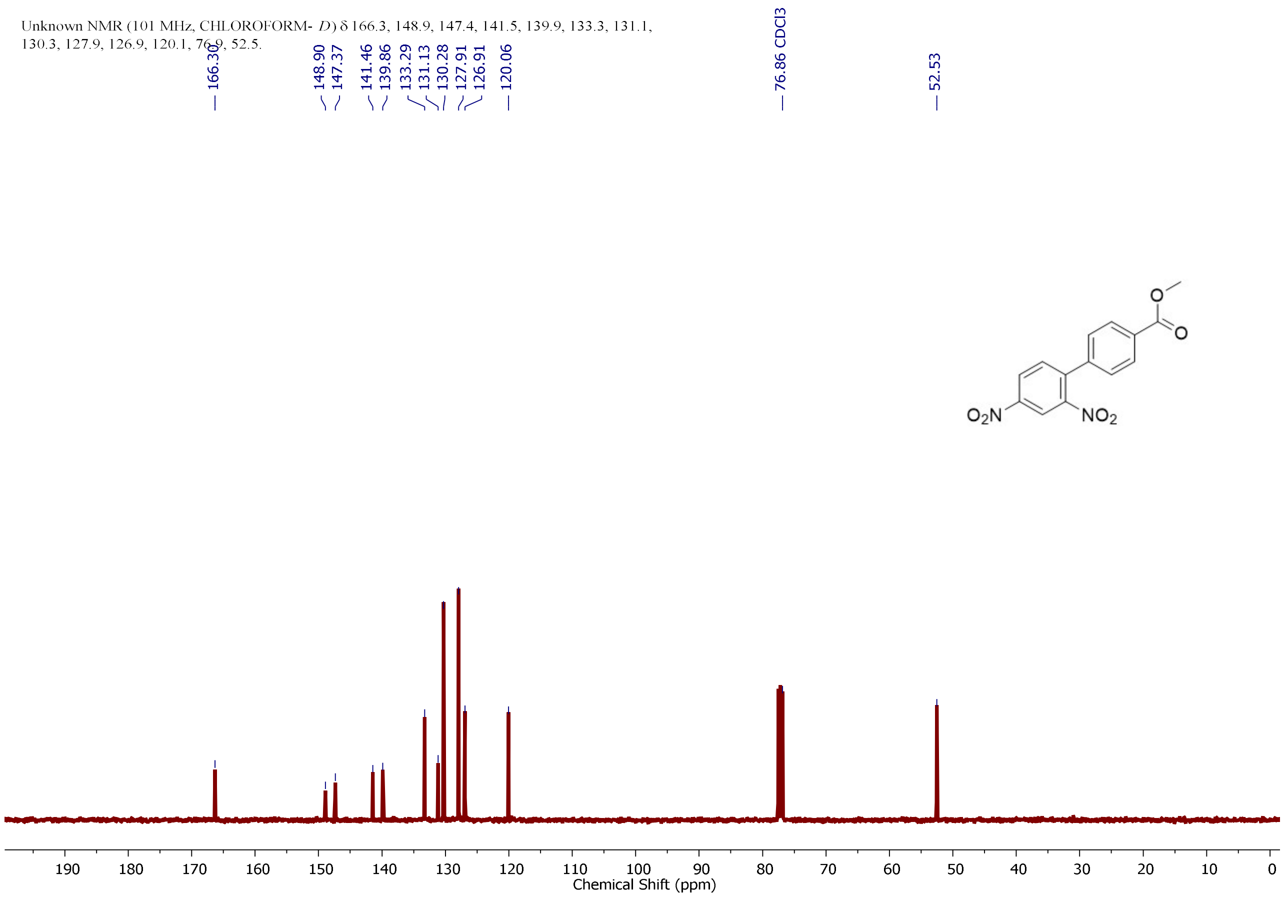

Figure S15b: ^{13}C NMR spectrum of S15.

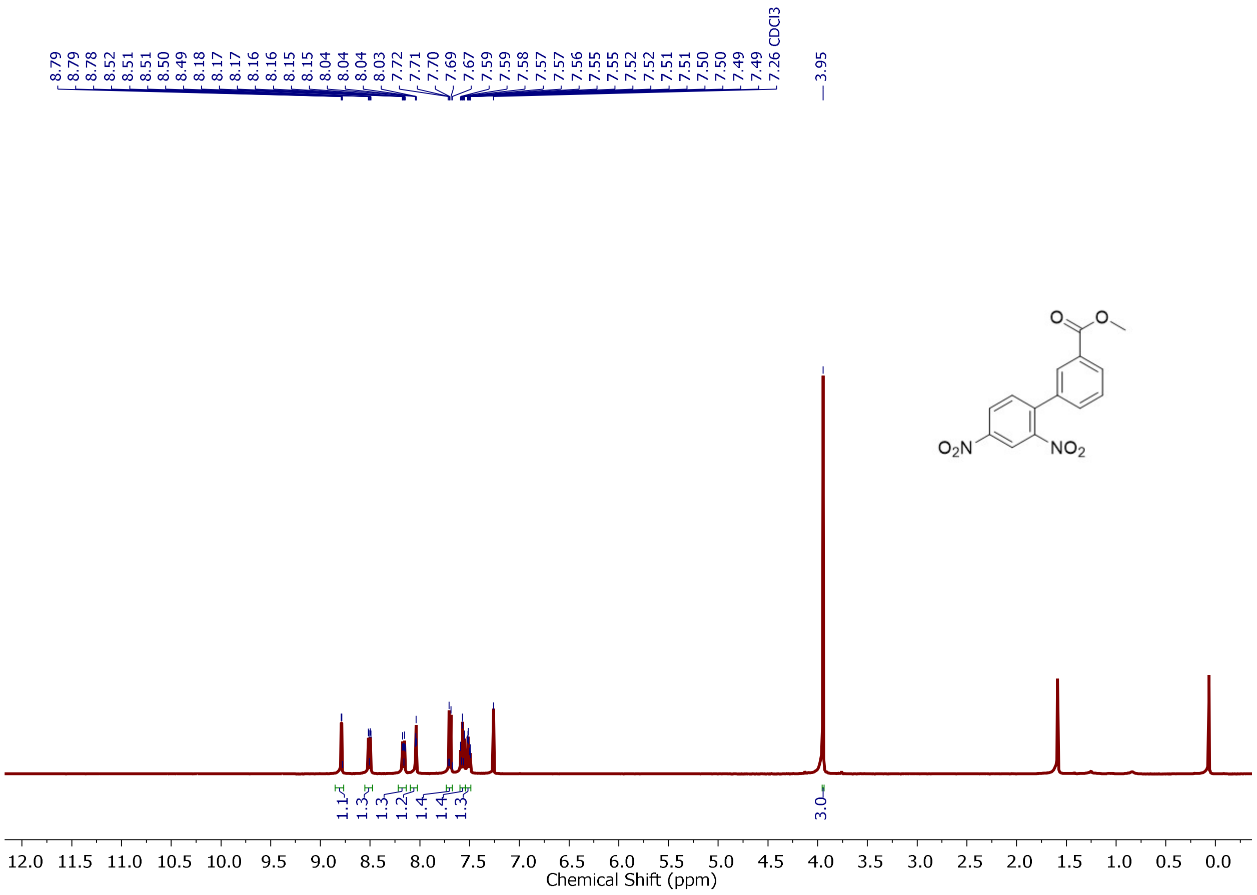

Figure S16a: ^{1}H NMR spectrum of S16.

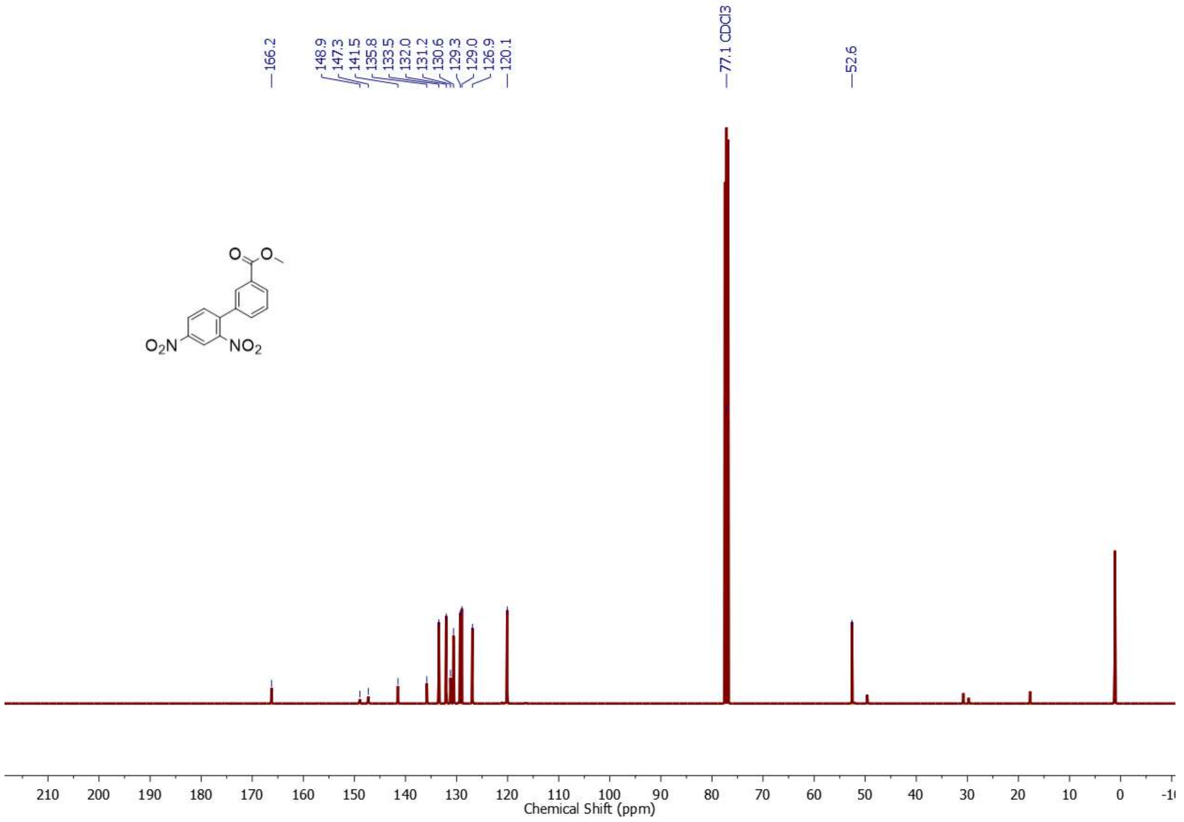

Figure S16b: ^{13}C NMR spectrum of S16.

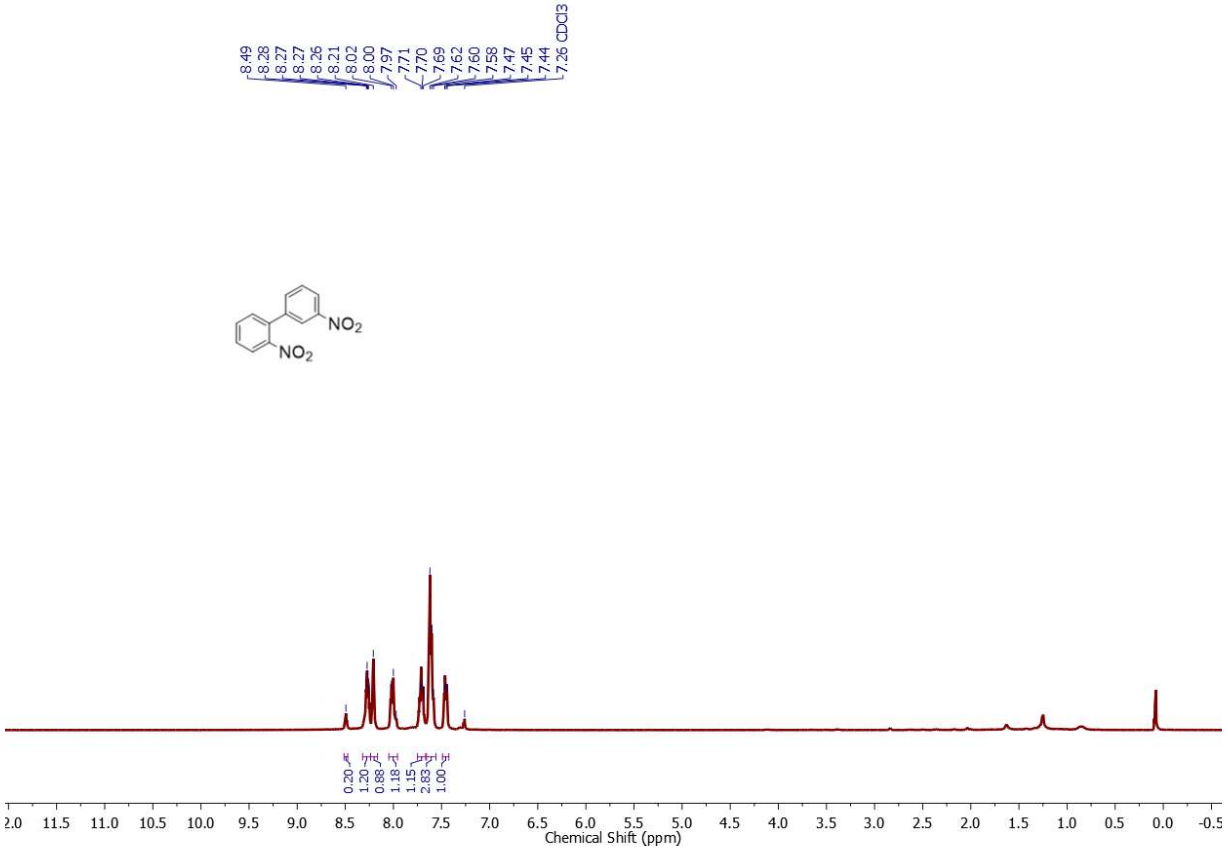

Figure S17a: ^1H NMR spectrum of S3.

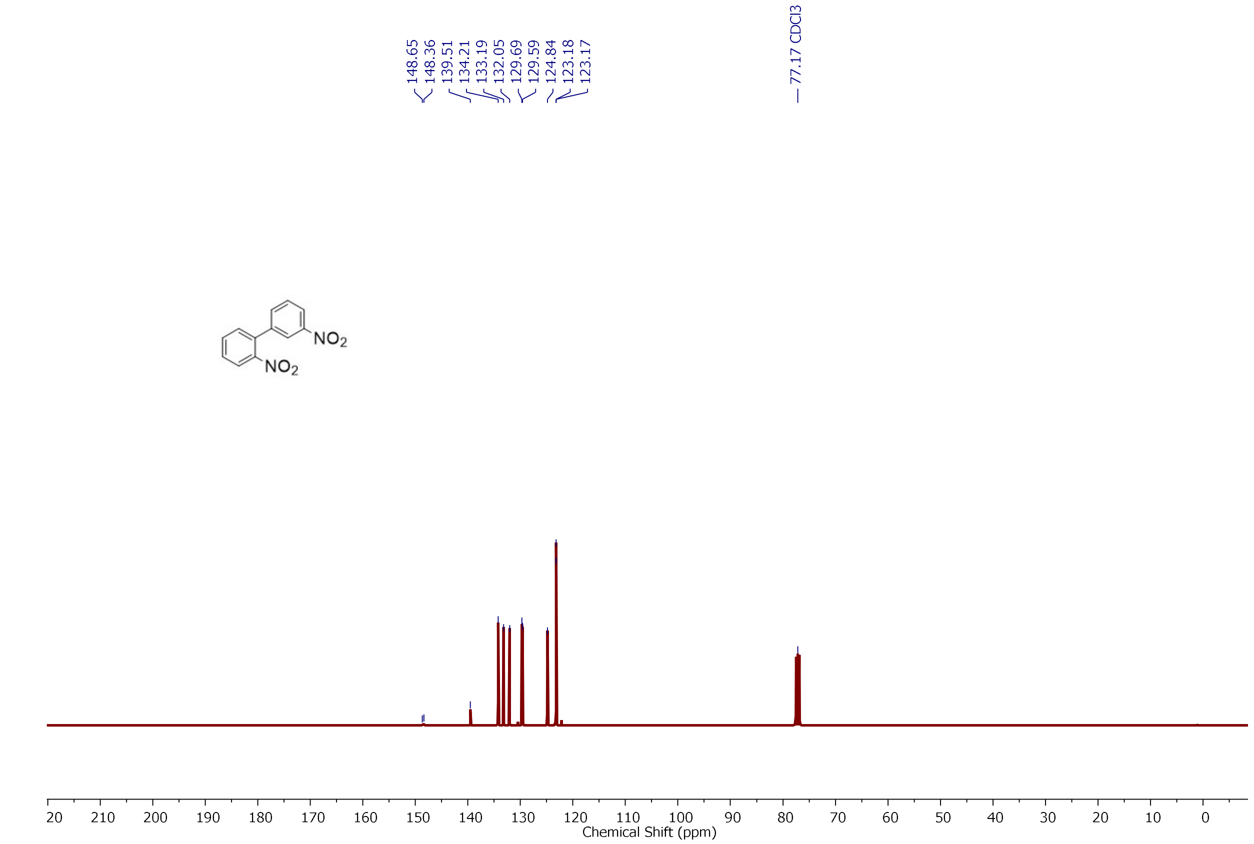

Figure S17b: ^{13}C NMR spectrum of S3.

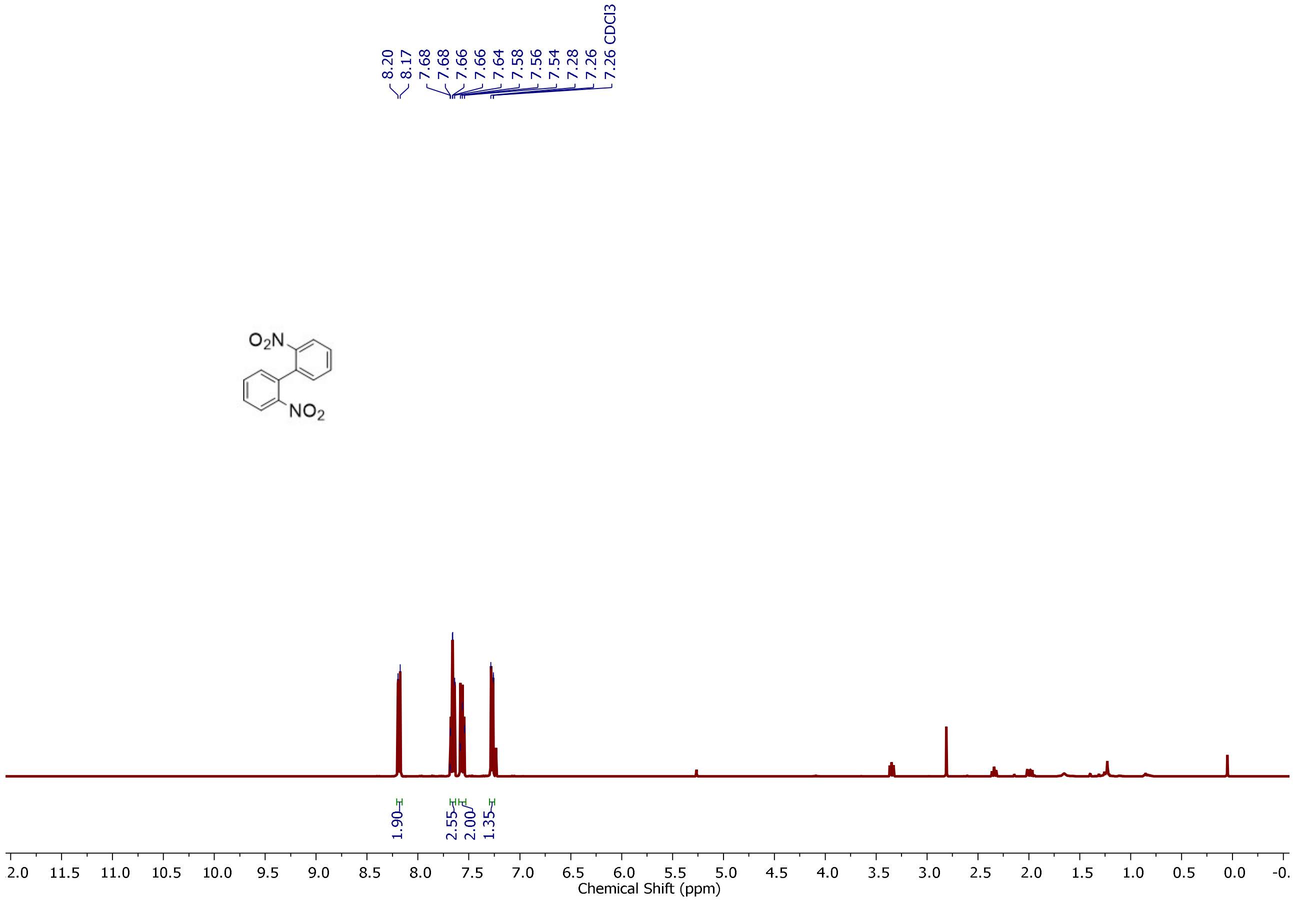

Figure S18a: ^{1}H NMR spectrum of S18.

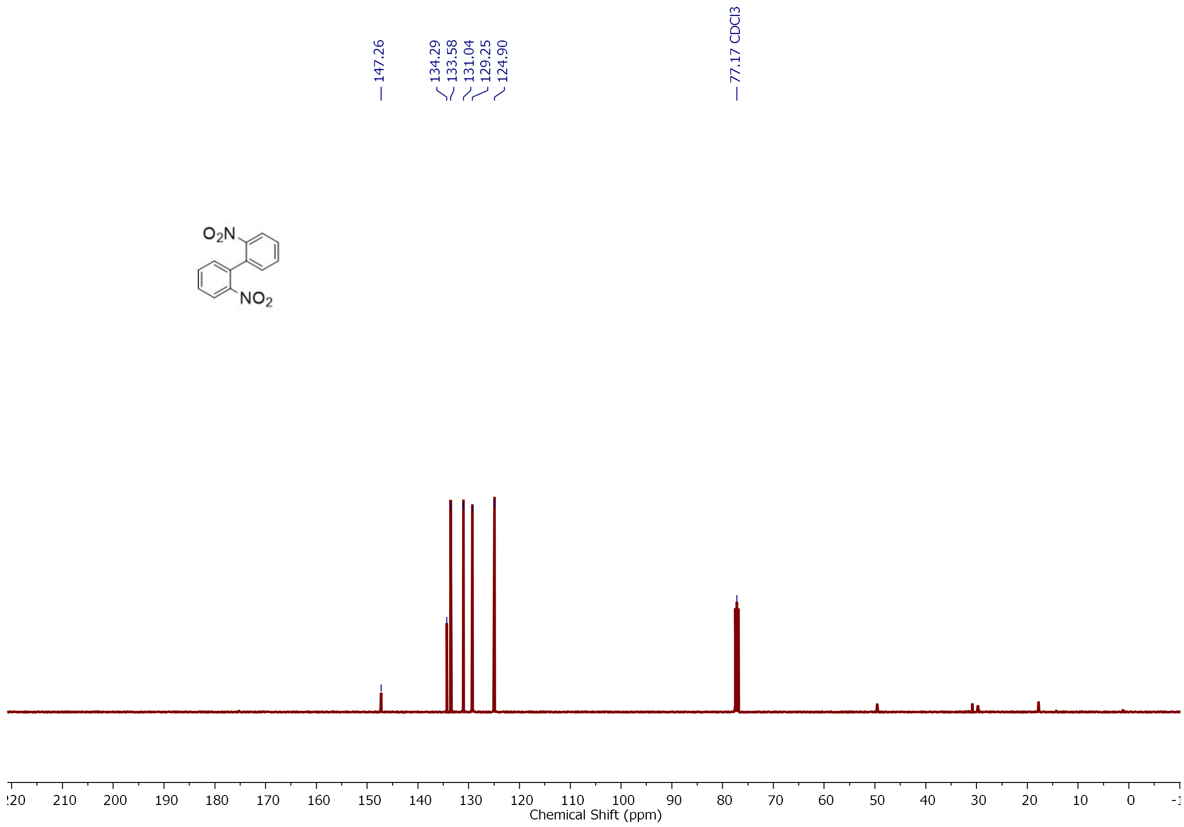

Figure S18b: ^{13}C NMR spectrum of S18.

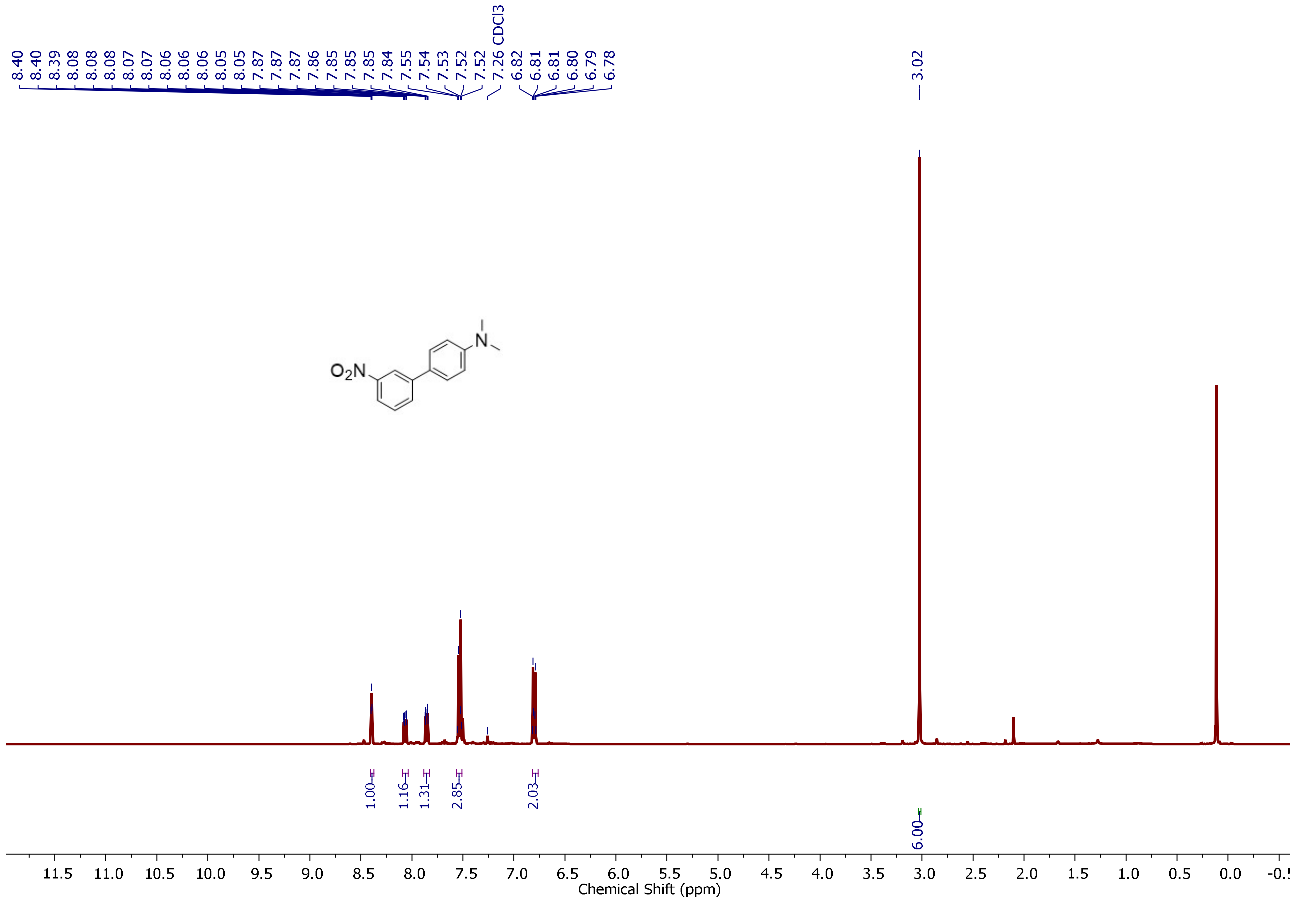

Figure S19a: ^{1}H NMR spectrum of S19.

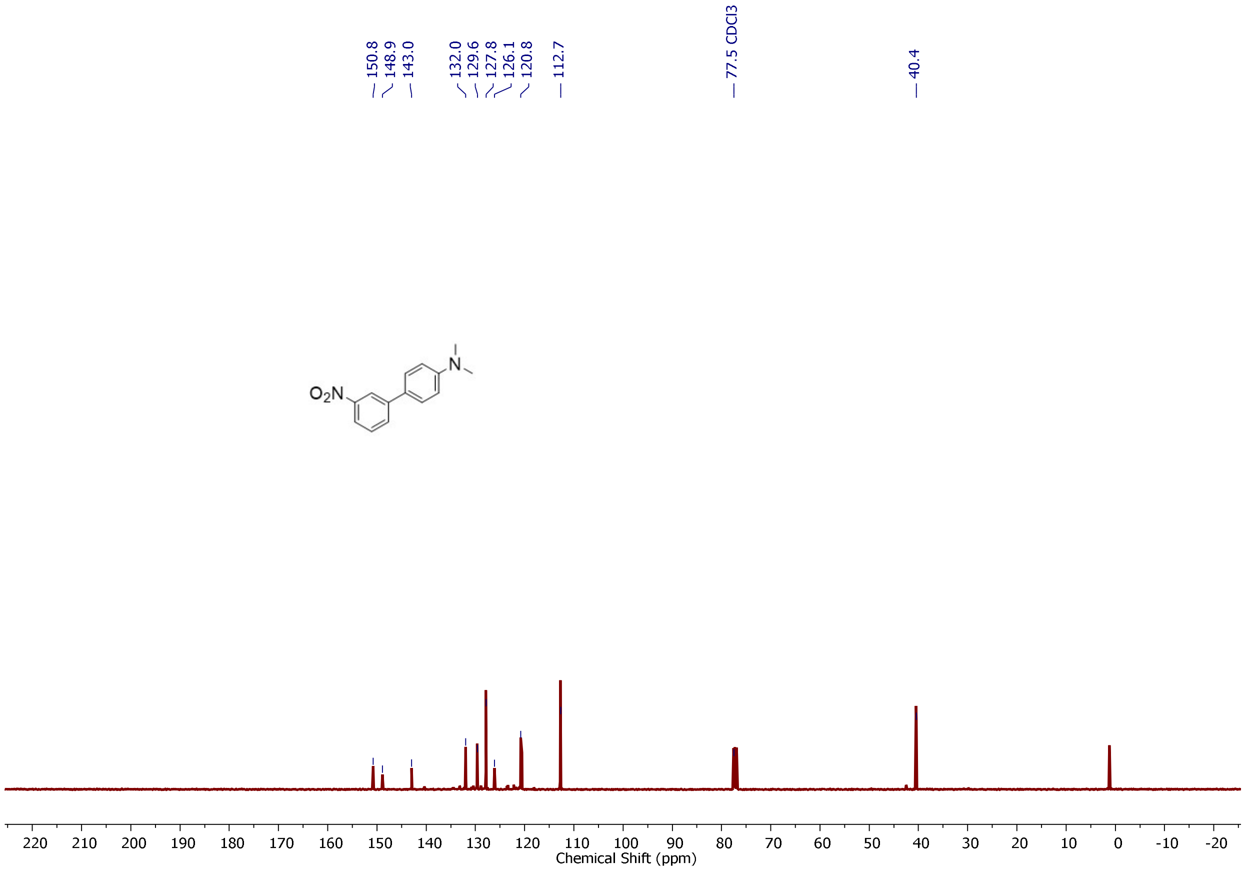

Figure S19b: ^{13}C NMR spectrum of S19.

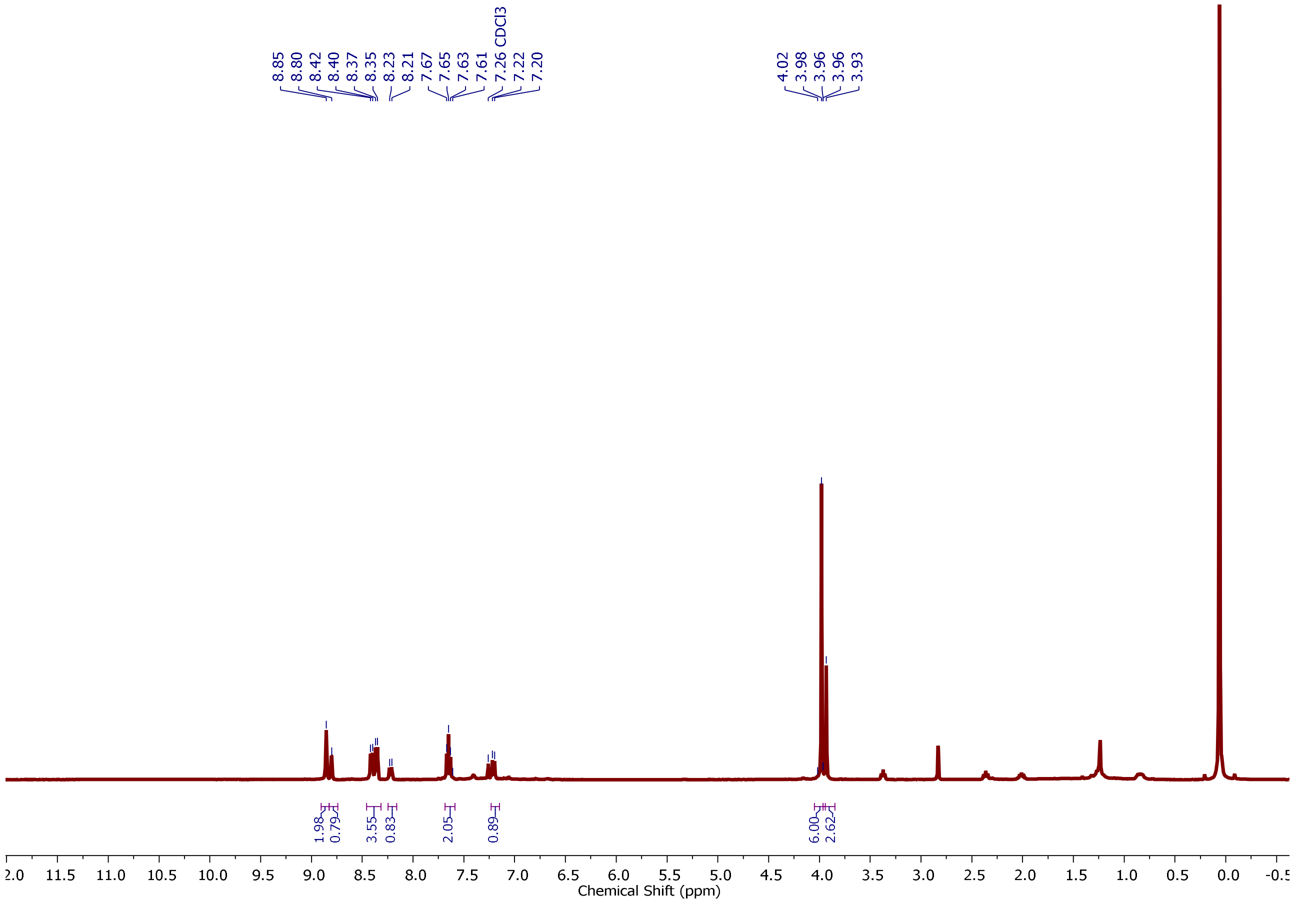

Figure S20a: ^{1}H NMR spectrum of S20.

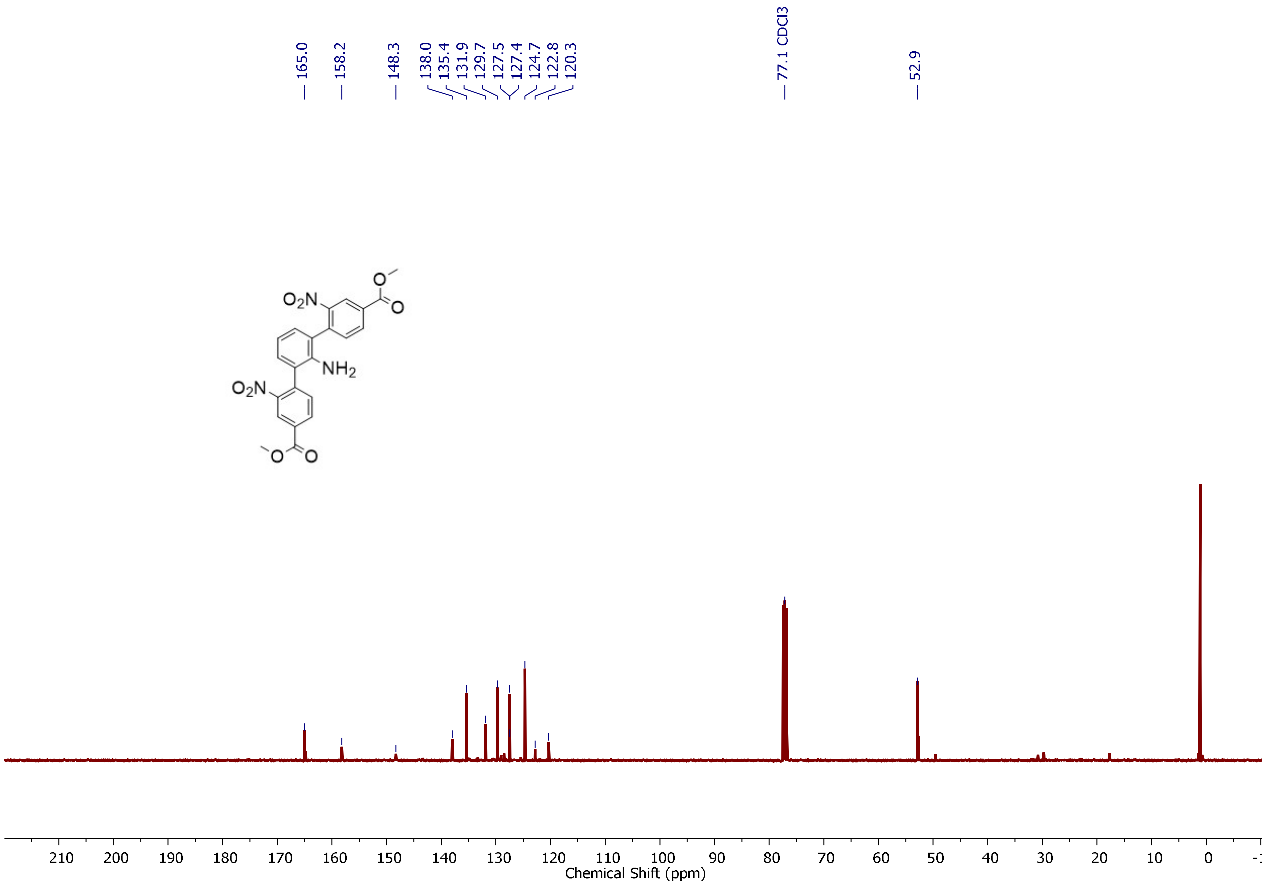

Figure S20b: ^{13}C NMR spectrum of S20.
